# *MCTS1* as a Novel Prognostic Biomarker and Its Correlation With Immune Infiltrates in Breast Cancer

**DOI:** 10.3389/fgene.2022.825901

**Published:** 2022-02-28

**Authors:** Mei Deng, Chao Xiong, Zhuo-Kai He, Qiong Bin, Jing-Zhi Song, Wei Li, Jie Qin

**Affiliations:** ^1^ Department of Radiation Oncology, Affiliated Hospital of Guilin Medical University, Guilin, China; ^2^ Department of Information, Affiliated Hospital of Guilin Medical University, Guilin, China; ^3^ Department of Nuclear Medicine, Affiliated Hospital of Guilin Medical University, Guilin, China

**Keywords:** MCTS1, breast cancer, biomarker, prognosis, methylation, immune infiltration, nomogram, bioinformatics

## Abstract

Multiple copies in T‐cell lymphoma‐1 (MCTS1) plays an important role in various cancers; however, its effects on patient prognosis and immune infiltration in breast cancer remain unclear. In this study, the expression profiles and clinical information of patients with breast cancer were obtained from the Cancer Genome Atlas (TCGA) database. Using the Wilcoxon rank-sum test, the *MCTS1* expression levels were compared between breast cancer and normal breast tissues. Functional enrichment analyses were performed to explore the potential signaling pathways and biological functions that are involved. Immune cell infiltration was assessed using single-sample gene set enrichment analysis. The UALCAN and MethSurv databases were used to analyze the methylation status of the *MCTS1*. The Kaplan-Meier method and Cox regression analysis were used to identify the prognostic value of *MCTS1*. A nomogram was constructed to predict the overall survival (OS) rates at one-, three-, and five-years post-cancer diagnosis. *MCTS1* was overexpressed in breast cancer and significantly associated with the M pathological stage, histological type, PAM50, and increased age. *MCTS1* overexpression contributes to a significant decline in OS and disease-specific survival. Multivariate Cox analysis identified *MCTS1* as an independent negative prognostic marker of OS. The OS nomogram was generated with a concordance index of 0.715. Similarly, the hypomethylation status of *MCTS1* is also associated with poor prognosis. Functional enrichment analysis indicated that the enriched pathways included the reactive oxygen species signaling pathway, MYC targets, interferon alpha response, immune response regulating signaling pathway, and leukocyte migration. Moreover, the overexpression of *MCTS1* was negatively correlated with the levels of immune cell infiltration of natural killer cells, CD8^+^ T cells, effector memory T cells, and plasmacytoid dendritic cells. Therefore, *MCTS1* maybe a novel prognostic biomarker.

## Introduction

Breast cancer is one of the most common malignancy that threatens the health of women worldwide (Sung et al., 2021). Despite significant improvements in the diagnosis and treatment of breast cancer, there are still a large number of patients who have disease and are resistant to therapy or relapse after treatment. Thus, there is an urgent need for the improvement of current diagnostic and therapeutic methods. The treatment decisions and prognosis for patients with breast cancer mainly depend on the tumor, node, and metastasis (TNM) staging system and molecular subtyping (Perou et al., 2000; Cserni et al., 2018). However, clinical outcomes vary even for patients with the same tumor stage and molecular subtyping, receiving similar treatment regimens. This indicates that the current staging system is not sufficient to accurately predict prognosis, nor does it accurately represent the biological heterogeneity of breast cancer patients. Other characteristics that are intrinsic to both the tumor cells and patients may influence the clinical outcomes. Studying these characteristics as prognostic markers could aid the current TNM staging system, may help predict clinical outcomes more accurately, provide better personalized treatments, and develop novel therapies for breast cancer.

Multiple copies in T‐cell lymphoma‐1 (*MCTS1*, also known as *MCT-1*), which was first observed to be amplified in T‐cell lymphoma in 1998, is an oncogene located on chromosome Xq22-24 (Prosniak et al., 1998). Previous studies have reported that constitutive expression of *MCTS1* leads to the transformation of NIH3T3 mouse fibroblasts and MCF-10A mammary epithelial cells and reduced doubling time, shortened the G1 phase of the cell cycle, and enhanced the activities of cyclin D1, cyclin-dependent kinase 4 (CDK4) and CDK6 (Dierov et al., 1999; Hsu et al., 2005). *MCTS1* is involved in several vital processes, including DNA damage responses (Nandi et al., 2007), translation reinitiation through its binding partner DENR (Schleich et al., 2014; Ahmed et al., 2018), regulation of mitotic progression and spindle assembly (Shih et al., 2012), and excretion of lactic acid as a product of anaerobic glycolysis (Halestrap, 2013). High expression of *MCTS1* increases the level of CD44, a tumor stem cell marker (Yan et al., 2015). Additionally, *MCTS1* is related to the acquisition of the invasive phenotype of oral cancer cells and lung adenocarcinoma cells through the modulation of the epithelial-mesenchymal transition process, and by the regulation of *E2F1* expression and the c‐Myc signaling pathway (Gao et al., 2021; Huang et al., 2021). In malignant pleural mesothelioma cells, *MCTS1* plays an important role in maintaining metabolic homeostasis and promoting malignancy. Elevation of *MCTS1* expression also increases the xenograft tumorigenicity of MCF-7 breast cancer cells by promoting angiogenesis and inhibiting apoptosis (Levenson et al., 2005). *MCTS1* expression may serve as a potential prognostic predictor for Luminal A, Luminal B, and triple-negative breast cancer subtypes of breast cancer (Weng et al., 2019; Tian et al., 2020). These findings indicate that *MCTS1* has multiple functions in various malignant tumors. However, due to the heterogeneity of breast cancer, the tumorigenic effects, clinical significance, and tumor immunology of abnormal expression of *MCTS1* in breast cancer are currently not fully understood.

In this study, we aimed to investigate and understand the relationship between *MCTS1* expression and its clinicopathological and prognostic significance, underlying molecular mechanisms, and immune cell infiltration in breast cancer using bioinformatics, which may help clinicians refine treatment and improve outcomes of patients with breast cancer.

## Materials and Methods

### Data Collection and Processing

We collected the mRNA expression profiles and clinical data of patients with breast cancer from the Cancer Genome Atlas (TCGA) database^1^ and the Genotype Tissue Expression Project (GTEx)^2^ database (n = 179). The level 3 HTSeq-FPKM format data were normalized as transcripts per million reads (TPM). We also obtained the RNA-sequencing data in TPM format from the UCSC Xena^3^ database and the GTEx database for pan-cancer analysis.

### Pathological Sample Collection

A total of 31 samples of paraffin-embedded breast cancer tissues and their matched paracancerous tissues were collected between April 2016 and April 2018 at the Pathology Department of Affiliated Hospital of Guilin Medical University. This study was approved by the medical ethics committees of Affiliated Hospital of Guilin Medical University (Approval Number: QTLL202136) and was conducted in line with the Declaration of Helsinki.

### Immunohistochemistry

Immunohistochemistry (IHC) staining was carried out as previously described (DeRycke et al., 2009). Briefly, tumor tissues and paracancerous tissues were fixed in 10% formalin, paraffin-embedded, sliced into 4∼6 μm sections, and placed onto slides. After deparaffinization, rehydration and microwave antigen retrieval, the slides were incubated with MCTS1 (Abcam, Cat #ab238825) antibody at 1:800 dilution at 4°C overnight. Afterwards, the slides were incubated with secondary antibody at room temperature for 30 min and stained with DAB substrate, followed by haematoxylin counterstaining.

### Differentially Expressed Gene Analysis

According to the median score of *MCTS1* expression, patients with breast cancer in TCGA were divided into high and low *MCTS1* expression groups. The R package DESeq2 was used to perform the differentially expressed gene (DEG) analysis between these two groups (Love et al., 2014), and adjusted *p* value <0.05, and |log_2_-fold-change (FC)|>1 were set as the thresholds of DEGs. The correlation between the expression of the top 10 DEGs and *MCTS1* was evaluated using Spearman’s correlation analysis.

### Functional Enrichment Analysis

Functional enrichment analyses, including Gene Ontology (GO) and Kyoto Encyclopedia of Genes and Genomes (KEGG) analysis, were implemented for the DEGs using the R package GOplot (version 1.0.2) (Walter et al., 2015). Gene set enrichment analysis (GSEA) was carried out using the R package clusterProfiler (Subramanian et al., 2005; Yu et al., 2012), and an adjusted *p* value <0.05 and false discovery rate (FDR) < 0.25 were regarded as statistically significantly enriched function or pathway terms.

### Protein-Protein Interaction Network Analysis

Based on the DEGs, a protein-protein interaction (PPI) network was established using the online STRING database^4^ with a confidence score >0.7 and other parameters left as default, and the PPI network was visualized using the Cytoscape software (version 3.5.1)^5^ (Szklarczyk et al., 2019). Subsequently, CytoHubba, a plugin in the Cytoscape software, was utilized to identify the top 10 hub genes of these DEGs (Chin et al., 2014).

### Immune Infiltration Analysis

A total of 24 immune cells were used to calculate the level of immune infiltration, and the relative enrichment score of these immune cells in breast cancer was assessed by single-sample GSEA, which was accomplished using the R package GSVA (Bindea et al., 2013). The correlation between the expression of *MCTS1* and these immune cells was investigated using the Spearman’s correlation analysis, and the differences in the level of immune infiltration between the high and low *MCTS1* expression groups were evaluated using the Wilcoxon rank-sum test.

### DNA Methylation Analysis

To explore the underlying mechanism of *MCTS1* on breast cancer, the UALCAN database^6^ was used to investigate the status of *MCTS1* promoter methylation (Chandrashekar et al., 2017). Additionally, the prognostic value of the *MCTS1* methylation level was assessed using the MethSurv database^7^, which is an online tool for multivariable survival analysis based on DNA methylation data (Modhukur et al., 2018).

### Survival Analysis

The Kaplan-Meier method with the log-rank test was used for survival analysis, and the cut-off value was set at the median expression level of *MCTS1*. Univariate and multivariate Cox regression analyses were used to assess the effect of clinical variables on patient outcomes. The prognostic variables *p* < 0.1 in the univariate Cox regression analysis were entered into multivariate Cox regression analysis. The R package ggplot2 was used to visualize the forest map.

### Construction and Validation of the Nomogram

To predict the overall survival probability, a nomogram was established based on independent prognostic factors in multivariate Cox analysis. Calibration plots were then used to assess the performance of the nomogram, and the concordance index (C-index) was used to quantify the discrimination of the nomogram. The nomogram and calibration plots were created using the R package RMS (version 5.1–4)^8^. The time-dependent receiver-operating characteristic (ROC) curve was performed to evaluate the predictive accuracy using the timeROC package.

### Statistical Analysis

All statistical analyses were conducted using R (version 3.6.3)^9^. The Wilcoxon rank-sum test and paired sample *t*-test were used to assess statistical significance for the expression of *MCTS1* in the non-paired and paired tissues, respectively. The Wilcoxon rank-sum test and logistic regression were used to assess the correlations between clinical features and *MCTS1* expression. All of the tests were two-sided, and *p* values <0.05, were regarded as statistically significant.

## Results

### Patient Characteristics

Our cohort included 1,065 breast cancer patients with clinical information and RNA-sequencing data, of which 110 patients had matched adjacent normal tissue samples, which were retrieved from TCGA. In addition, to increase the sample size of normal breast tissues, we obtained the gene expression data of normal breast tissues (n = 179) from the GTEx database. The clinicopathological characteristics of patients with breast cancer are shown in Supplementary Table S1.

### Elevated Expression of *MCTS1* in Breast Cancer

**FIGURE 1:**
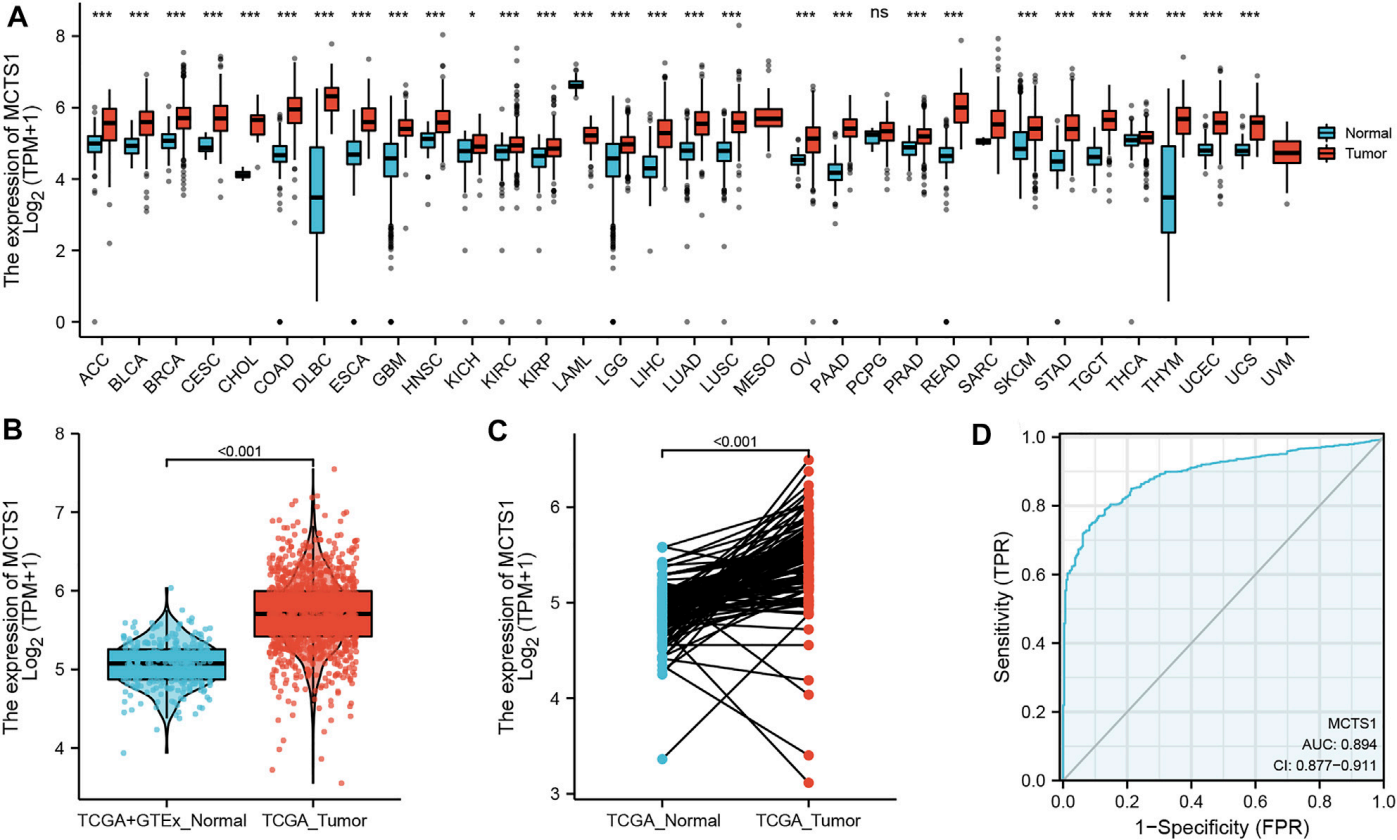
Expression levels of *MCTS1* in different types of tumors and breast cancer. Expression of *MCTS1* **(A)** in different types of tumors compared with normal tissues in TCGA and GTEx databases, **(B)** in breast cancer and non-matched normal tissues in the TCGA and GTEx databases, and **(C)** in breast cancer and matched normal tissues in TCGA database. **(D)** ROC curves for classifying breast cancer versus normal breast tissues in the TCGA database. TCGA, The Cancer Genome Atlas; GTEx, Genotype Tissue Expression Project; ROC, receiver operating characteristic. ^∗^ *p* < 0.05, ^∗∗^ *p* < 0.01, and ^∗∗∗^ *p* < 0.001.

The pan-cancer analysis showed that the expression of *MCTS1* was highly expressed in most types of cancers, such as adrenocortical carcinoma, bladder urothelial carcinoma, cervical squamous cell carcinoma, adenocarcinoma, and cholangiocarcinoma (Figure 1A). The expression of *MCTS1* was significantly higher in breast cancer samples than in normal breast tissues (*p* < 0.001) (Figure 1B). In addition, *MCTS1* was highly expressed in 110 paired breast cancer tissues (*p* < 0.001) (Figure 1C). Furthermore, the ROC curve indicated that *MCTS1* expression had good predictive power with an area under the curve (AUC) of 0.894 (95% confidence interval [CI] = 0.877–0.911) to discriminate breast cancer tissues from normal tissues (Figure 1D).

**FIGURE 2:**
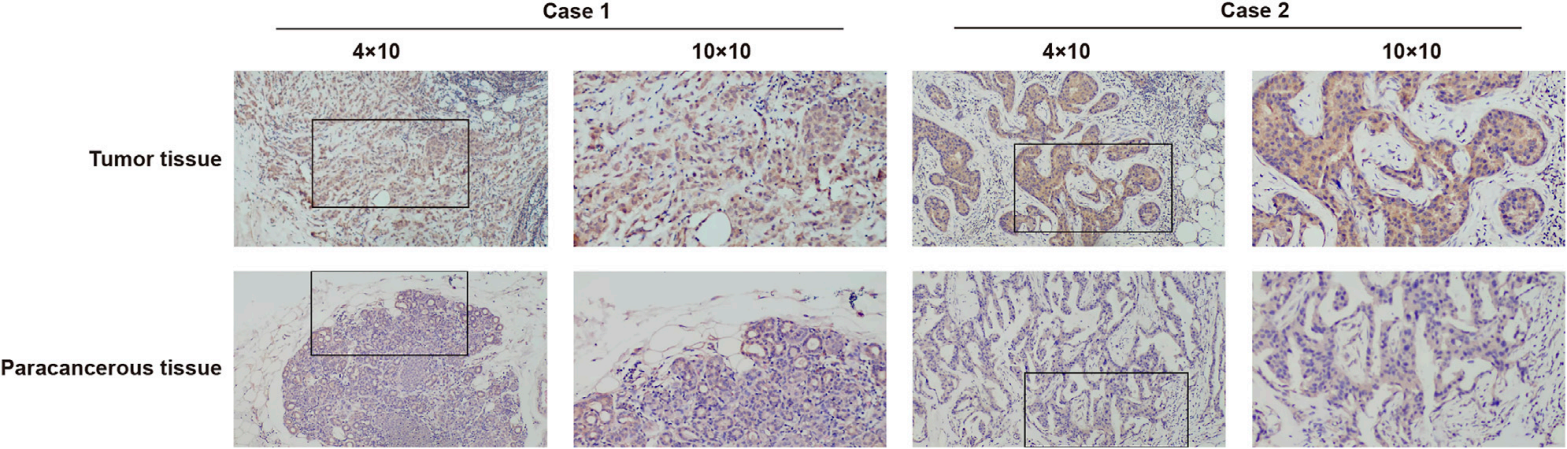
Representative images of MCTS1 expression in breast cancer tissues and their matched paracancerous tissues. Original magnifications 40× and 100× (inset panels).

To further identify the expression of MCTS1, IHC staining was carried out on a cohort comprising 31 cases of primary breast cancer tissues paired with noncancerous tissues. There were 31 females with a mean age of 50.4 years (range 26–78 years) involved in this cohort. The expression of MCTS1 in 80.6% (25/31) of breast cancer tissues was upregulated *via* IHC. Representative images are presented in Figure 2.

### Associations Between *MCTS1* Expression and Clinicopathologic Variables

**FIGURE 3:**
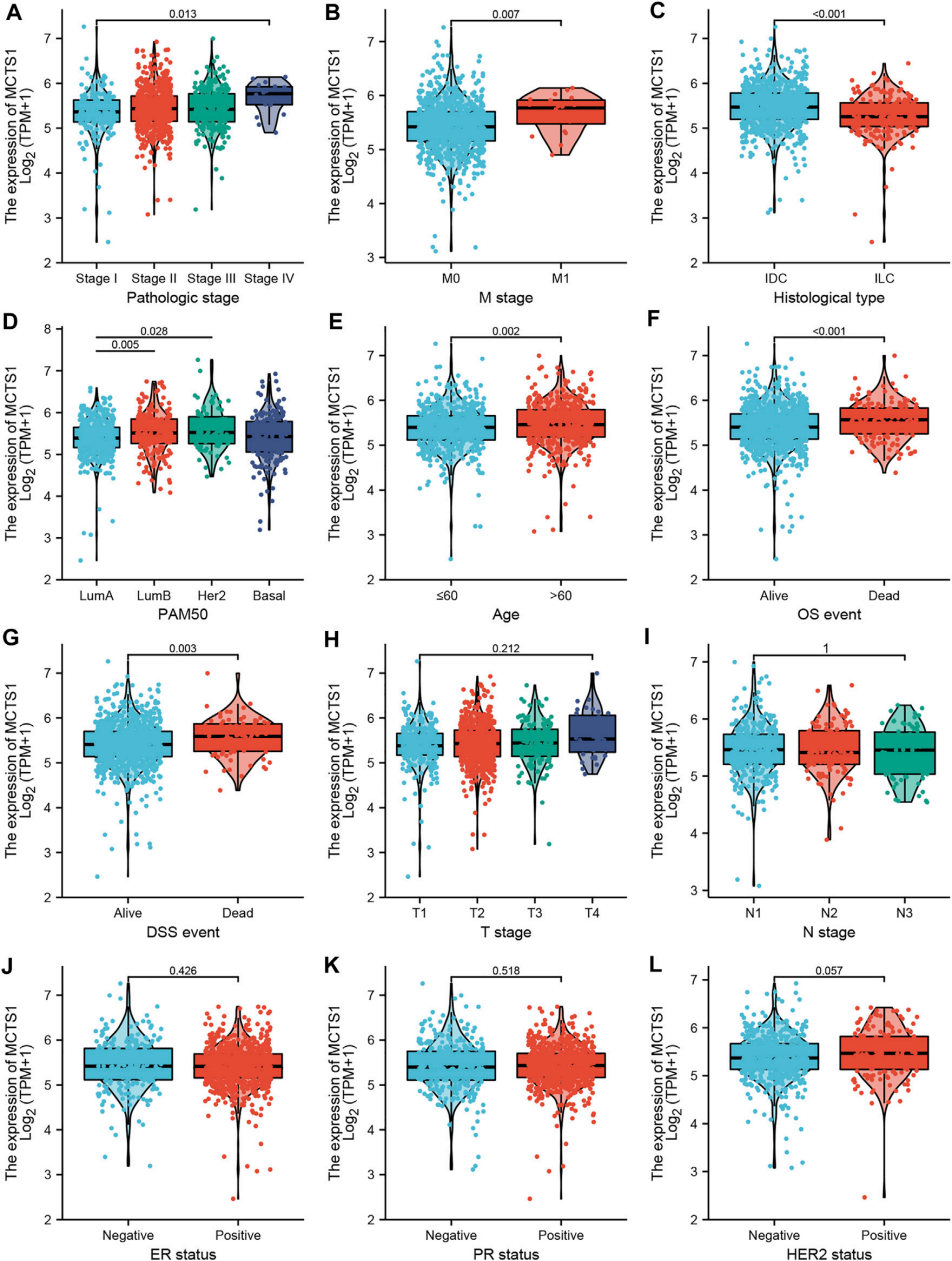
Associations between *MCTS1* expression and clinicopathological characteristics. Data are shown for **(A)** pathological stage; **(B)**M stage; **(C)** histological type; **(D)** PAM50; **(E)** age; **(F)** OS event; **(G)** DSS event; **(H)** T stage; **(I)** N stage; **(J)** ER status; **(K)** PR status; and **(L)** HER2 status. IDC, infiltrating ductal carcinoma; ILC, infiltrating lobular carcinoma; LumA, Luminal A; LumB, Luminal B; OS, overall survival; DSS, disease-specific survival; ER, estrogen receptor; PR, progesterone receptor; HER2, human epidermal growth factor receptor 2.

As shown in Table 1 and Figure 3, high expression of *MCTS1* was significantly associated with pathologic stage (stage IV vs. stage I, *p* = 0.013), M stage (*p* = 0.007), histological type (*p* < 0.001), PAM50 (Luminal B vs. Luminal A, *p* = 0.005; human epidermal growth factor receptor 2 [HER2] vs. Luminal A, *p* = 0.028), age (*p* = 0.002), overall survival (OS) (*p* < 0.001), and disease-specific survival (DSS) events (*p* = 0.003). Meanwhile, the results of the univariate logistic regression analyses showed that there were certain clinicopathological differences between the groups with high and low expression of *MCTS1*, including M stage (odds ratio [OR] = 3.048, 95% CI = 1.170–9.435, *p* = 0.032), age (OR = 1.281, 95% CI = 1.006–1.632, *p* = 0.045), race (OR = 0.667, 95% CI = 0.496–0.894, *p* = 0.007), PAM50 (OR = 1.463, 95% CI = 1.143–1.874, *p* = 0.003), and histological type (OR = 0.439, 95% CI = 0.317–0.605, *p* < 0.001) (Table 2).

**TABLE 1:** Clinicopathological characteristics of high- and low-*MCTS1* expression groups.

Characteristic	Levels	Low expression of *MCTS1*	High expression of *MCTS1*	*p* value
n		532	533	
Age, median (IQR)		57.5 (48, 65)	59 (49, 69)	**0.048**
Age, n (%)	≤60	310 (29.1%)	278 (26.1%)	0.052
	>60	222 (20.8%)	255 (23.9%)	
T stage, n (%)	T1	146 (13.7%)	129 (12.1%)	0.292
	T2	306 (28.8%)	309 (29.1%)	
	T3	65 (6.1%)	72 (6.8%)	
	T4	13 (1.2%)	22 (2.1%)	
N stage, n (%)	N0	259 (24.8%)	248 (23.7%)	0.654
	N1	165 (15.8%)	184 (17.6%)	
	N2	61 (5.8%)	55 (5.3%)	
	N3	36 (3.4%)	38 (3.6%)	
M stage, n (%)	M0	448 (49.3%)	441 (48.5%)	**0.043**
	M1	5 (0.6%)	15 (1.7%)	
Pathologic stage, n (%)	Stage I	99 (9.5%)	81 (7.8%)	0.050
	Stage II	296 (28.4%)	310 (29.8%)	
	Stage III	122 (11.7%)	116 (11.1%)	
	Stage IV	4 (0.4%)	14 (1.3%)	
Race, n (%)	Asian	20 (2%)	40 (4.1%)	**0.005**
	Black or African American	83 (8.5%)	96 (9.8%)	
	White	392 (40.2%)	345 (35.3%)	
Histological type, n (%)	Infiltrating Ductal Carcinoma	343 (35.8%)	414 (43.2%)	**< 0.001**
	Infiltrating Lobular Carcinoma	132 (13.8%)	70 (7.3%)	
PR status, n (%)	Negative	180 (17.7%)	158 (15.6%)	0.404
	Indeterminate	2 (0.2%)	2 (0.2%)	
	Positive	331 (32.6%)	343 (33.8%)	
ER status, n (%)	Negative	119 (11.7%)	118 (11.6%)	0.970
	Indeterminate	1 (0.1%)	1 (0.1%)	
	Positive	394 (38.7%)	384 (37.8%)	
HER2 status, n (%)	Negative	294 (41%)	254 (35.4%)	0.139
	Indeterminate	5 (0.7%)	7 (1%)	
	Positive	71 (9.9%)	86 (12%)	
PAM50, n (%)	Normal	30 (2.8%)	10 (0.9%)	**< 0.001**
	Luminal A	294 (27.6%)	257 (24.1%)	
	Luminal B	79 (7.4%)	123 (11.5%)	
	Her2	34 (3.2%)	48 (4.5%)	
	Basal	95 (8.9%)	95 (8.9%)	
Menopause status, n (%)	Pre	126 (13.2%)	98 (10.3%)	0.130
	Peri	18 (1.9%)	21 (2.2%)	
	Post	338 (35.4%)	355 (37.1%)	
Anatomic neoplasm subdivisions, n (%)	Left	267 (25.1%)	286 (26.9%)	0.284
	Right	265 (24.9%)	247 (23.2%)	
Radiation therapy, n (%)	No	225 (23.1%)	207 (21.3%)	0.601
	Yes	271 (27.9%)	269 (27.7%)	
OS event, n (%)	Alive	479 (45%)	439 (41.2%)	**< 0.001**
	Dead	53 (5%)	94 (8.8%)	
DSS event, n (%)	Alive	498 (47.6%)	467 (44.6%)	**0.002**
	Dead	27 (2.6%)	54 (5.2%)	

Abbreviations: ER, estrogen receptor; PR, progesterone receptor; HER2, human epidermal growth factor receptor 2; OS, overall survival; DSS, disease-specific survival.

Bold values denote two-sided *p* < 0.05.

**TABLE 2:** Associations of *MCTS1* expression with clinicopathological characteristics of patients (n = 1,065).

Characteristics	Total (N)	Odds ratio (OR)	*p* value
T stage (T3&T4 vs. T1&T2)	1,062	1.244 (0.897–1.729)	0.192
N stage (N1&N2&N3 vs. N0)	1,046	1.104 (0.866–1.408)	0.423
M stage (M1 vs. M0)	909	3.048 (1.170–9.435)	**0.032**
Pathologic stage (Stage III &Stage IV vs. Stage I &Stage II)	1,042	1.042 (0.786–1.382)	0.773
Age (>60 vs. ≤60)	1,065	1.281 (1.006–1.632)	**0.045**
Race (White vs. Asian &Black or African American)	976	0.667 (0.496–0.894)	**0.007**
ER status (Positive vs. Negative)	1,015	0.983 (0.735–1.315)	0.907
PR status (Positive vs. Negative)	1,012	1.181 (0.909–1.534)	0.214
HER2 status (Positive vs. Negative)	705	1.402 (0.983–2.006)	0.063
PAM50 (Luminal B&Her2&Basal vs. Luminal A)	1,025	1.463 (1.143–1.874)	**0.003**
Menopause status (Post vs. Pre &Peri)	956	1.271 (0.956–1.691)	0.099
Anatomic neoplasm subdivisions (Right vs. Left)	1,065	0.870 (0.684–1.107)	0.257
Radiation therapy (Yes vs. No)	972	1.079 (0.838–1.390)	0.556
Histological type (Infiltrating Lobular Carcinoma vs. Infiltrating Ductal Carcinoma)	959	0.439 (0.317–0.605)	**<0.001**

Abbreviations: ER, estrogen receptor; PR, progesterone receptor; HER2, human epidermal growth factor receptor 2.

Bold values denote two-sided *p* < 0.05.

### Identification of DEGs in Breast Cancer and PPI Network Analysis

**FIGURE 4:**
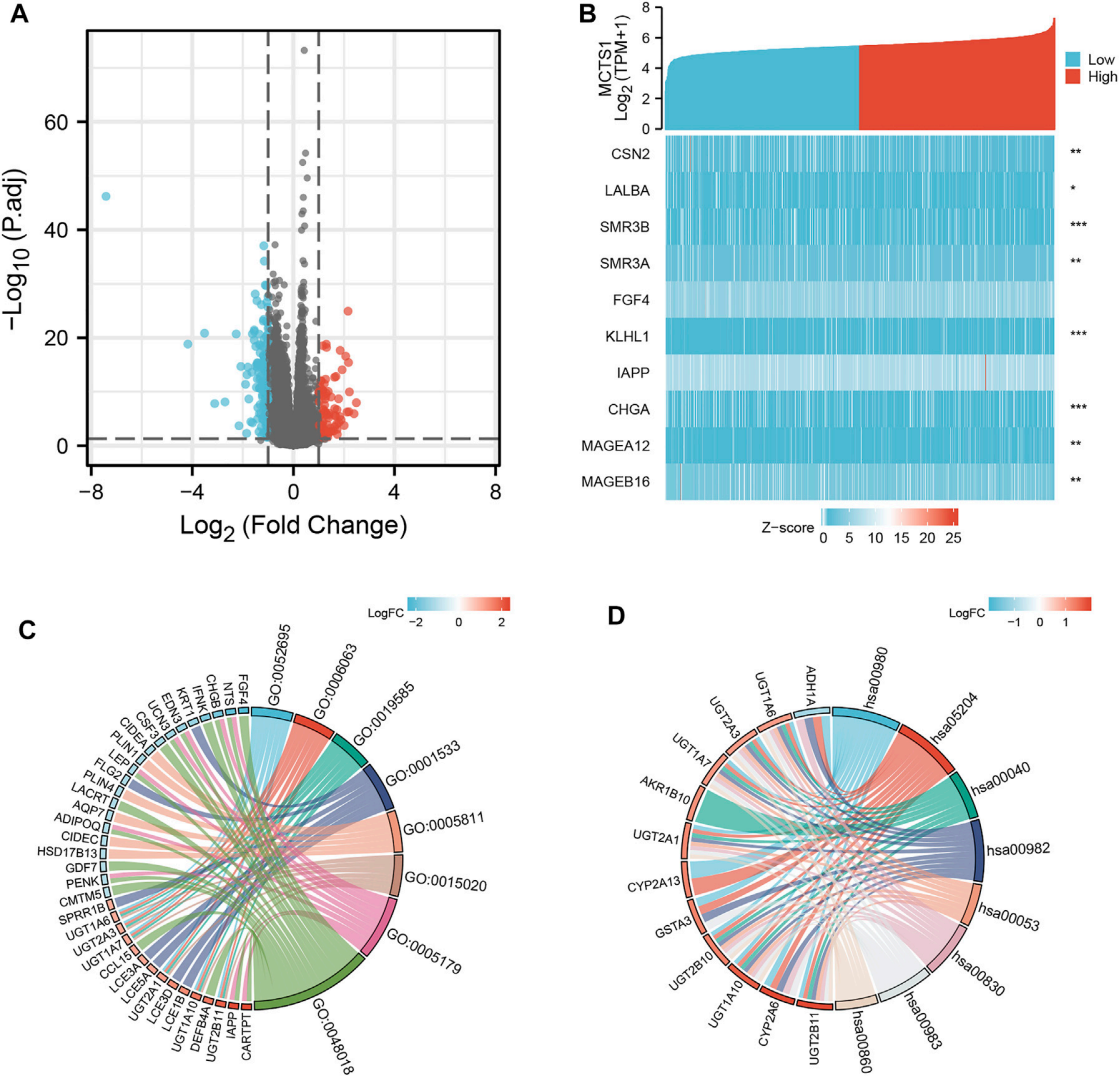
*MCTS1*-related differentially expressed genes (DEGs) and functional enrichment analysis of *MCTS1* in breast cancer using GO and KEGG. **(A)** Volcano plot of DEGs. Blue and red dots indicate the significantly down-regulated and up-regulated DEGs, respectively. **(B)** Heatmap of correlation between *MCTS1* expression and the top 10 DEGs. **(C)** GO analysis of DEGs. **(D)** KEGG analysis of DEGs. GO, Gene Ontology; KEGG, Kyoto Encyclopedia of Genes and Genomes; DEGs, differentially expressed genes. ^∗^ *p* < 0.05, ^∗∗^ *p* < 0.01, and ^∗∗∗^ *p* < 0.001.

A total of 226 genes were differentially expressed between the groups with high and low expression levels of *MCTS1*, including 89 upregulated DEGs (39.4%) and 137 downregulated DEGs (60.6%) (adjusted *p* value <0.05, |Log_2_-FC| > 1) (Figure 4A and Supplementary Table S2). Next, the relationship between the top 10 DEGs (including *CSN2*, *LALBA*, *SMR3B*, *SMR3A*, *FGF4*, *KLHL1*, *IAPP*, *CHGA*, *MAGEA12*, and *MAGEB16*) and *MCTS1* are presented in Figure 4B. To explore the potential interactions among all identified DEGs, we constructed a PPI network using the online STRING tool, and then identified the hub genes. As shown in Supplementary Figure S1, the network of the DEGs was complex, and the top 10 hub genes were *CYP2A6*, *CYP2A13*, *ADH1B*, *CD19*, *PLIN1*, *LCE3A*, *LCE3D*, *LCE1B*, *CASP14*, and *LEP*.

### Functional Enrichment Analysis Including GO, KEGG, and GSEA Analysis

**FIGURE 5:**
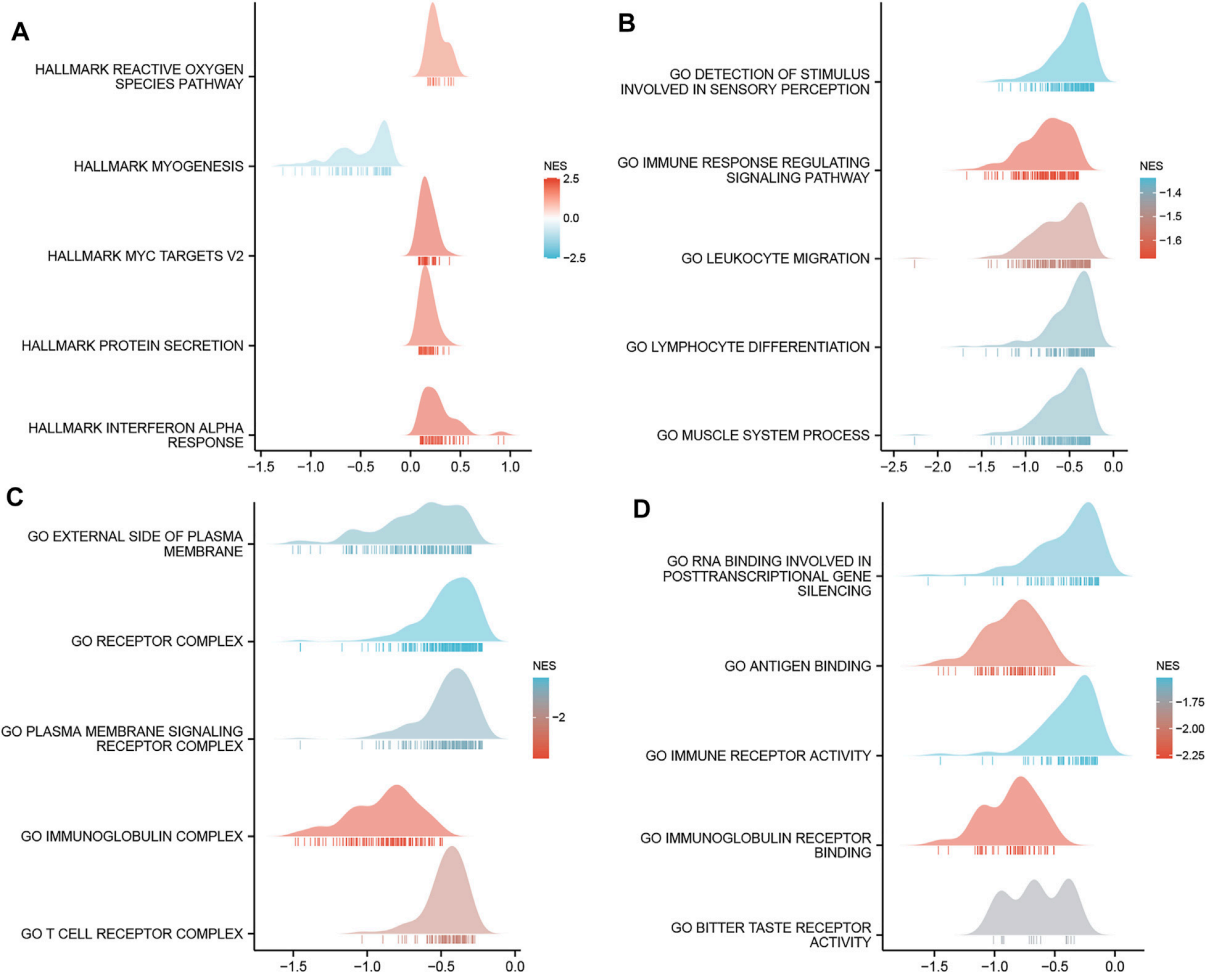
Gene set enrichment analysis (GSEA) of DEGs. **(A)** GSEA analysis of the Hallmark gene sets deposited in MSigDB. **(B)** GSEA analysis of the bp of Gene Ontology gene sets downloaded from MSigDB. **(C)** GSEA analysis of the cc of Gene Ontology gene sets downloaded from MSigDB. **(D)** GSEA analysis of the mf of Gene Ontology gene sets downloaded from MSigDB. GSEA, gene set enrichment analysis; DEGs, differentially expressed genes; MSigDB, Molecular Signatures database; NES, normalized enrichment score.

GO enrichment analysis, including biological processes, cellular compositions, and molecular functions revealed that DEGs were enriched in different GO terms such as glucuronate metabolic process, glucuronate metabolic process, cornified envelope, glucuronosyltransferase activity, and hormone activity (Figure 4C and Supplementary Table S3). Additionally, KEGG pathway analysis showed that significantly DEGs-enriched pathways included pentose and glucuronate interconversions, chemical carcinogenesis, metabolism of xenobiotics by cytochrome P450, and drug metabolism (Figure 4D and Supplementary Table S4). Subsequently, GSEA was applied between the high- and low-*MCTS1* expression groups, and more immune-related biological processes were found to be significantly enriched in the low *MCTS1* expression group, suggesting that the high expression of *MCTS*1 conferred a decreased immune phenotype in breast cancer (Figures 5A–D).

### Correlation Between Methylation and Expression of *MCTS1*

**FIGURE 6:**
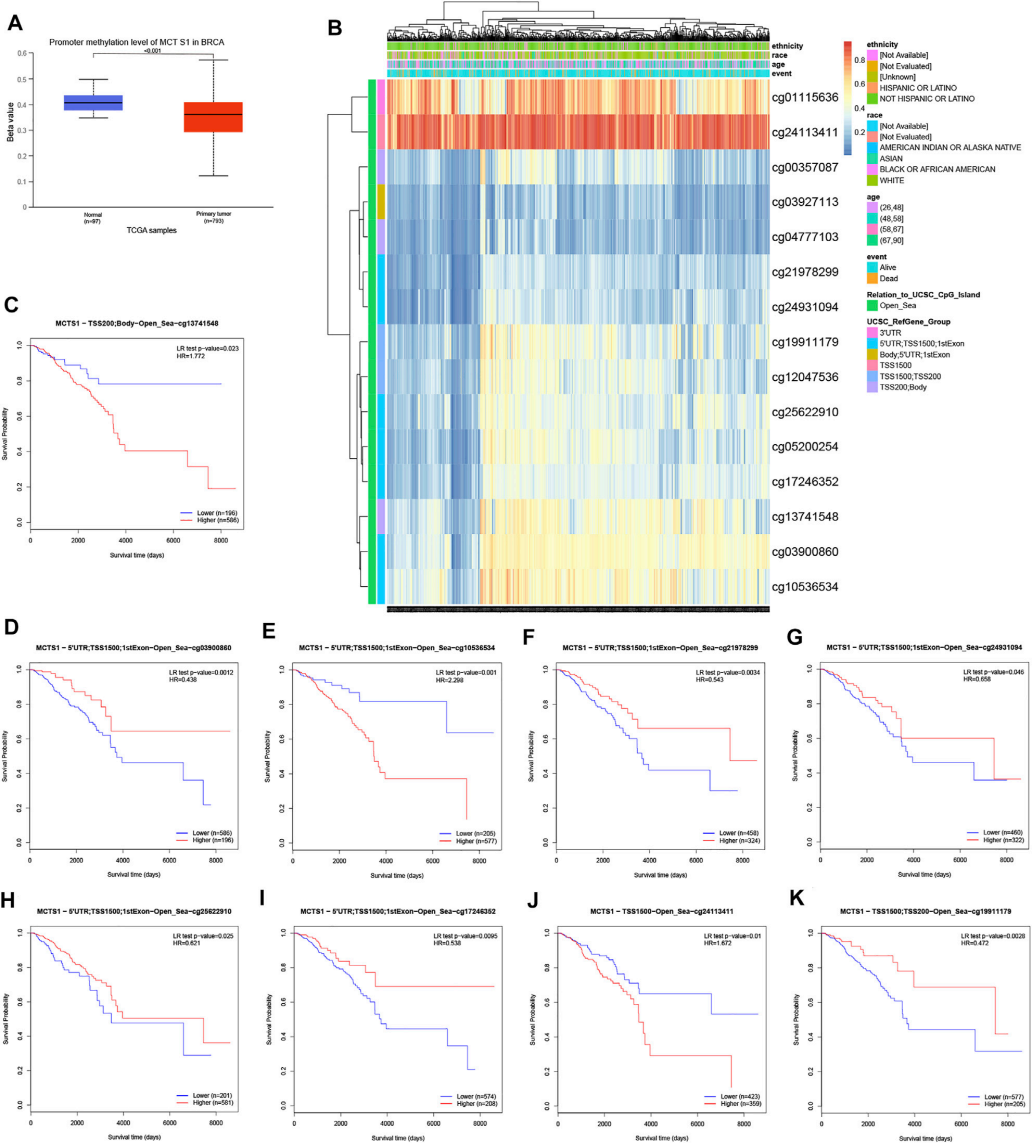
DNA methylation level of *MCTS1* and its effect on prognosis of patients with breast cancer. **(A)** The promoter methylation level of *MCTS1* in breast cancer was obtained from the UALCAN database. **(B)** Correlation between *MCTS1* mRNA expression level and methylation level. **(C–K)** Kaplan-Meier survival curves for several methylation sites of *MCTS1*.

To clarify the underlying mechanisms of *MCTS1* overexpression in breast cancer tissues, we also investigated the correlation between *MCTS1* expression levels and methylation status using online tools. First, we observed that the breast cancer tumor tissues exhibited a significantly lower level of DNA methylation at the promoter than that in the normal breast tissues using the UALCAN database (*p* < 0.001) (Figure 6A). We found that the majority of methylation sites in the DNA sequences of *MCTS1* were hypomethylated in breast cancer, and the degree of methylation was correlated with patient outcomes (i.e., patients with low *MCTS1* methylation had poorer overall survival than patients with high *MCTS1* methylation) (Figure 6B). Finally, several methylation sites were indicative of poor prognosis, including *cg03900860*, *cg21978299*, *cg24931094*, *cg25622910*, *cg17246352*, and *cg19911179* (Figures 6C–K).

### Correlation Between *MCTS1* Expression and Immune Infiltration

**FIGURE 7:**
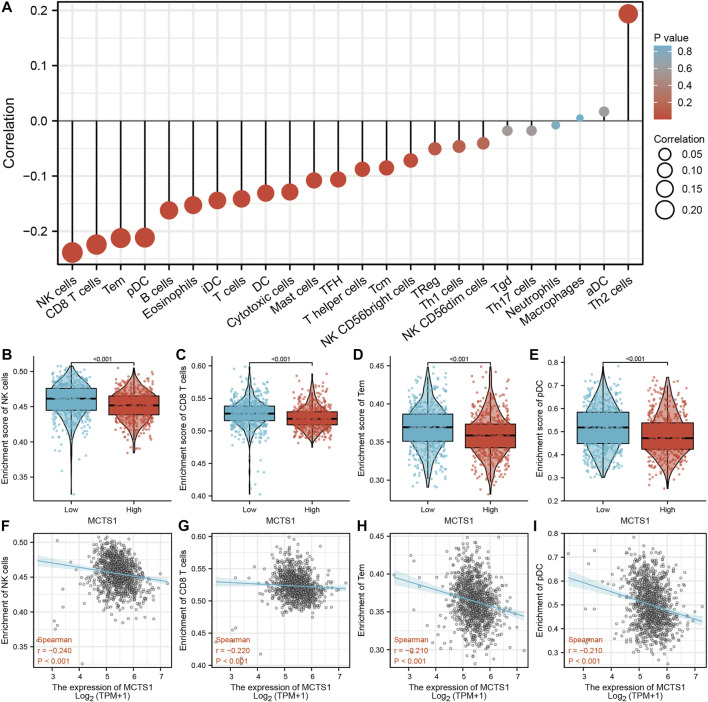
Correlation of *MCTS1* expression with immune infiltration level in breast cancer. **(A)** Correlation between *MCTS1* expression and relative abundance of 24 types of immune cell. The size of dot corresponds to the absolute Spearman’s correlation coefficient values. **(B–E)** Comparison of immune infiltration levels of immune cells (including NK cells, CD8^+^ T cells, TEM cells, and pDCs) between the high- and low-*MCTS1* expression groups. **(F–I)** Correlations between the relative enrichment scores of immune cells (including NK cells, CD8^+^ T cells, TEM cells, and pDCs) and the expression of *MCTS1*. NK cells, natural killer cells; TEM cells, effector T cells; pDCs, plasmacytoid dendritic cells.

The expression of *MCTS1* was significantly negatively correlated with the levels of immune cell infiltration of natural killer (NK) cells (*r* = –0.240, *p* < 0.001), CD8^+^ T cells (*r* = –0.220, *p* < 0.001), effector memory T (TEM) cells (*r* = –0.210, *p* < 0.001), and plasmacytoid dendritic cells (pDCs) (*r* = –0.210, *p* < 0.001) (Figure 7A). In addition, the enrichment scores of NK cells, CD8^+^ T cells, TEM cells, and pDCs in the *MCTS1* high expression group were markedly lower than those in the *MCTS1* low expression group (all *p* < 0.001) (Figures 7B–I).

### Prognostic Value of *MCTS1* in Breast Cancer

**FIGURE 8:**
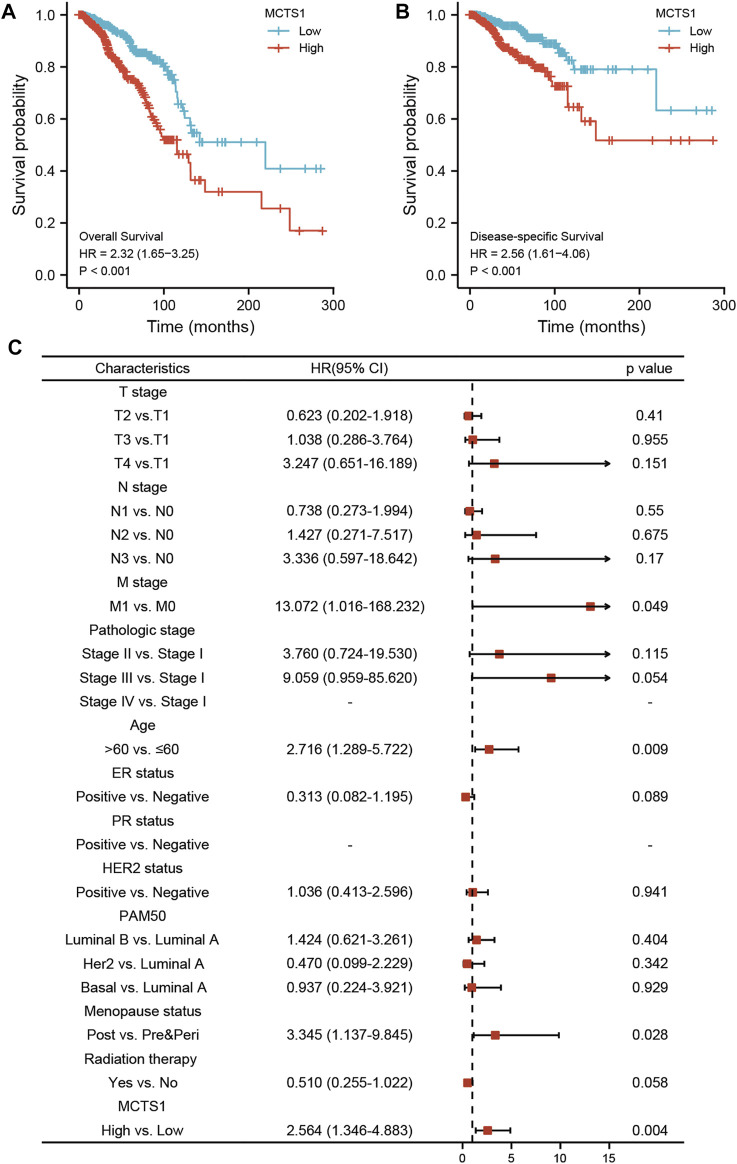
Prognostic values of *MCTS1* expression in patients with breast cancer evaluated by the Kaplan-Meier method. Overall survival **(A)** and disease-specific survival **(B)** for breast cancer patients with high versus low *MCTS1*. **(C)** Forest map based on multivariate Cox analysis for overall survival. HR, hazard ratio; CI, confidence interval. ER, estrogen receptor; PR, progesterone receptor; HER2, human epidermal growth factor receptor 2.

The correlation between *MCTS1* expression and the prognosis of patients with breast cancer was calculated using the Kaplan-Meier method. The median value of *MCTS1* expression was used as a cut-off score, and the patients were divided into high and low *MCTS1* expression groups. Compared with the low *MCTS1* expression group, both the OS and DSS of the high *MCTS1* expression group exhibited a significantly worse prognosis (OS: hazard ratio [HR] = 2.32, 95% CI = 1.65–3.25, *p* < 0.001; DSS: HR = 2.56, 95% CI = 1.61–4.06, *p* < 0.001) (Figures 8A,B). Additionally, in our hospital cohort, breast cancer patients with high *MCTS1* expression exhibited a low disease-free survival (*p* = 0.023) (Supplementary Figure S2). Next, the relationships between the expression of *MCTS1* and prognosis in different subgroups were evaluated.

**FIGURE 9:**
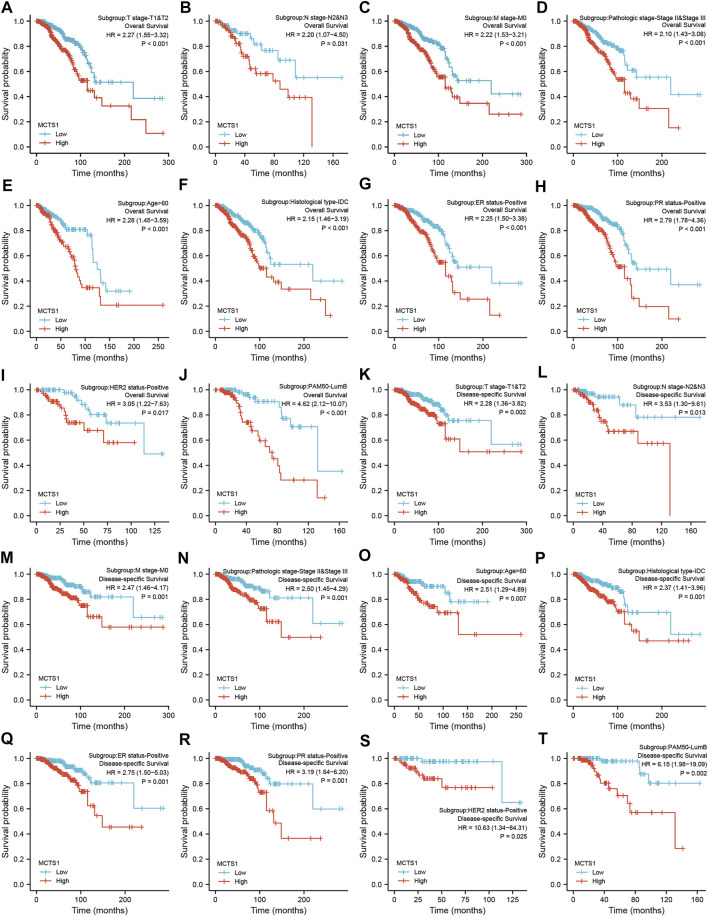
Prognostic values of *MCTS1* expression in patients with breast cancer evaluated by the Kaplan-Meier method in different subgroups. **(A–J)** OS survival curves of T1 and T2, N2 and N3, M0, stage II and III, age >60 years, IDC, ER positive, PR positive, HER2 positive, and Luminal B subgroups between high- and low-*MCTS1* patients with breast cancer. **(K–T)** DSS survival curves of T1 and T2, N2 and N3, M0, stage II and III, age >60 years, IDC, ER positive, PR positive, HER2 positive, and Luminal B subgroups between high- and low-*MCTS1* patients with breast cancer. OS, overall survival; DSS, disease-specific survival; ER, estrogen receptor; PR, progesterone receptor; HER2, human epidermal growth factor receptor 2; LumB, Luminal B; IDC, infiltrating ductal carcinoma.

Regardless of OS or DSS, the prognosis of patients with high *MCTS1* expression was notably more unfavorable in several subgroups, including T1 and T2, N2 and N3, M0, stage II and III, age >60 years, estrogen receptor (ER)-positive, progesterone receptor (PR)-positive, HER2-positive, Luminal B, and infiltrating ductal carcinoma (IDC) subgroups (all *p* < 0.05) (Figure 9).

Univariate and multivariate Cox regression analyses were conducted to identify prognostic indicators. The results of the multivariate analysis demonstrated that *MCTS1* expression (adjusted HR = 2.564, 95% CI = 1.346–4.883, *p* = 0.004), M stage (adjusted HR = 13.072, 95% CI = 1.016–168.232, *p* = 0.049), age (adjusted HR = 2.716, 95% CI = 1.289–5.722, *p* = 0.009), and menopausal status (adjusted HR = 3.345, 95% CI = 1.137–9.845, *p* = 0.028) were independent factors of OS in patients with breast cancer (Figure 8C and Supplementary Table S5). Similarly, for DSS, *MCTS1* expression (adjusted HR = 3.267, 95% CI = 1.723–6.195, *p* < 0.001), M stage (adjusted HR = 10.452, 95% CI = 1.230–88.798, *p* = 0.032), and T3 stage (adjusted HR = 0.219, 95% CI = 0.049–0.986, *p* = 0.048) proved to be prognostic indicators (Supplementary Table S6).

### Construction and Validation of a Nomogram Based on the Independent Factors

**FIGURE 10:**
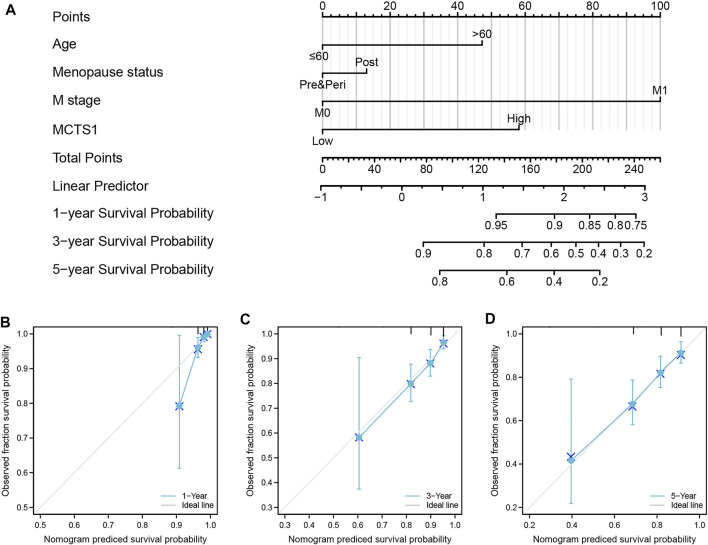
A nomogram and calibration curves for prediction of one-, three-, and five-year overall survival rates of patients with breast cancer. **(A)** A nomogram for prediction of one-, three-, and five -overall survival rates of patients with breast cancer. **(B–D)** Calibration curves of the nomogram prediction of one-, three-, and five-year overall survival rates of patients with breast cancer.

To predict the prognosis of patients with breast cancer, a nomogram based on the independent factors of OS was generated. On the nomogram, a higher total number of points was associated with a worse prognosis (Figure 10A). Additionally, calibration curves were used to assess the prediction efficacy of the nomogram (Figures 10B–D). The bootstrap corrected C-index of the nomogram was 0.715 (95% CI = 0.687–0.743), indicating that the model had a moderate predictive accuracy for OS of patients with breast cancer. Furthermore, the time-dependent ROC curve was applied to evaluate the discriminative ability of *MCTS1* expression and the constructed nomogram model, respectively (Supplementary Figures 3A–B). These results indicated that the nomogram was appropriate.

## Discussion

Due to the heterogeneity of breast cancer, the current pathological indicators (such as ER, PR, HER2, Ki67, and grade) that predict prognosis have some limitations. Thus, it is crucial to identify novel biomarkers to predict prognosis and enhance individualized therapies. In this study, we analyzed *MCTS1* expression in breast cancers using TCGA database and found that *MCTS1* was highly expressed in breast cancer compared to normal tissues. As a candidate oncogene, *MCTS1* was initially identified in human T-cell lymphoma, and the increased expression of *MCTS1* has been observed in several types of cancers, including diffuse large B-cell lymphomas (Dai et al., 2009), lung adenocarcinoma, oral cancer, neuroblastoma, breast cancer (Pinheiro et al., 20102010), bladder cancer (Afonso et al., 2015), clear cell renal cell carcinoma (Kim et al., 2015), gastrointestinal stromal tumors (de Oliveira et al., 2012), colorectal cancer (Pinheiro et al., 2008a), cervical cancer (Pinheiro et al., 2008b), malignant pleural mesothelioma (Mogi et al., 2013), and gastric cancer (Pinheiro et al., 2009). However, the expression levels of *MCTS1* in breast cancer is unclear, since contradicting results have been reported (Asada et al., 2003). Moreover, only a few studies have assessed the clinicopathological and prognostic significance of *MCST1* overexpression in certain types of tumors (Pinheiro et al., 2008a; Pinheiro et al., 2008b; Pinheiro et al., 2009; Pinheiro et al., 2010).

In this study, our results showed that high expression of *MCTS1* was associated with unfavorable clinicopathologic factors, such as stage IV, M1 stage, and Luminal B subtype, which is consistent with data from a previous study (Tian et al., 2020). However, Pinheiro et al. reported that upregulated *MCTS1* was more common in the basal-like subtype (Pinheiro et al., 2010). Moreover, our data indicated that elevated expression of *MCTS1* acts as an independent prognostic biomarker of poor OS and DSS in patients with breast cancer. Similarly, several previous studies have shown that *MCTS1* expression may be a potential prognostic indicator of reduced survival in patients with certain solid tumors, including lung cancer, oral cancer, bladder cancer, malignant pleural mesothelioma, gastrointestinal stromal tumors, clear cell renal cell carcinoma, and breast cancer subtypes Luminal A and B (Huang et al., 2021; Gao et al., 2021; Tian et al., 2020; Kim et al., 2015; de Oliveira et al., 2012; Choi et al., 2014; Dell’Anno et al., 2020). These results demonstrate that *MCTS1* can be used as an attractive and novel molecular target for effective cancer therapy.

Moreover, *MCTS1* has been shown to exert oncogenic effects on breast cancer progression by affecting multiple cancer-related signaling pathways, including modulation of c-Myc translation (Tian et al., 2020), IL-6/IL-6R signaling pathway (Weng et al., 2019), and Src/p190B signaling pathway (Wu et al., 2014). Nevertheless, these results do not fully elucidate the underlying mechanism of *MCTS1* in breast cancer, and the biological function and signaling pathway of *MCTS1* warrant further exploration. In the present study, we performed GSEA and found that significantly enriched pathways in the high *MCTS1* expression group included the reactive oxygen species (ROS) signaling pathway, MYC targets, interferon alpha response, immune response regulating signaling pathway, and leukocyte migration. A previous study reported that high expression of *MCTS1* induced the generation of ROS, which caused YY-1/EGFR/MnSOD signaling amplification and cancer cell invasion in lung cancer (Tseng et al., 2017). These findings require further experimental validation and may enrich the content of *MCTS1*-related biological functions in breast cancer.

DNA methylation is a common epigenetic mechanism of gene regulation, which generally silences gene expression (Lim and Maher, 2010). In this study, we further investigated underlying mechanism of *MCTS1* overexpression in breast cancer, and our data show that *MCTS1* overexpression may be related to its DNA hypomethylation. The hypomethylated level of *MCTS1* was associated with poor prognosis in patients with breast cancer. Data concerning the DNA methylation level of *MCTS1* in solid tumors are scarce, and there are conflicting data related to its role in breast cancer. To our knowledge, the DNA methylation level of *MCTS1* in breast cancer samples has been evaluated in only one study, where the investigators reported silencing of *MCTS1* in four out of the 19 breast cancer samples by hypermethylation of the 5′ upstream region (Asada et al., 2003). These inconclusive results may be because of differences in ethnic backgrounds and/or the small number of samples in the study.

Tumor cells grow in a complex microenvironment composed of cancer cells, immune cells, and stromal cells. Malignant tumor cells, including breast cancer cells, are usually surrounded by infiltrating immune cells. The prognostic value of tumor-infiltrating immune cells has been demonstrated in solid malignant tumors, which are affected by the type, density, and location of immune cells (Widowati et al., 2020). Additionally, infiltrating immune cells have also been shown to predict the response to neoadjuvant chemotherapy and immune checkpoint inhibition (ICI) treatment (Denkert et al., 2010; Havel et al., 2019). Hence, screening infiltrating immune cells in breast cancer could not only be aid in combination with ICI treatment, but also has potential predictive value for ICI therapy. Given that the high *MCTS1* expression group was enriched in leukocyte migration and immune response regulating signaling pathways, we then calculated the correlation between *MCTS1* expression and the levels of immune infiltration cells, which revealed that *MCTS1* overexpression was negatively associated with the infiltration of NK cells, CD8^+^ T cells, TEM cells, and pDCs. As innate immune cells, both activated pDCs and NK cells have been shown to arrest the growth of breast cancer cells (Wu et al., 2017; Widowati et al., 2020). The presence of CD8^+^ T cells has been associated with improved prognosis in patients with breast cancer (Mahmoud et al., 2011). These results indicated that *MCTS1* overexpression may affect the progression and prognosis of breast cancer by regulating the levels of infiltrating immune cells.

Although our current study offers new insights into the relationship between *MCTS1* expression and the prognostic value of patients with breast cancer, there are limitations that need to be considered. First, this study concerned only one dataset, which may have resulted in a selection bias. Second, because most of the data in this study were collected from the online databases, we were not able to obtain some important clinical information, such as the chemotherapy regimens patients received. Third, further investigation and rigorous experimental validation are needed for both *in vitro* and *in vivo* systems to elucidate the biological functions and underlying mechanisms of *MCTS1* in breast cancer.

In conclusion, this study revealed that high expression of *MCTS1* is an independent adverse prognostic factor in breast cancer, and is strongly associated with aggressive clinical features and unfavorable immune infiltration. Our findings suggest that *MCTS1* could be used as a novel prognostic biomarker for predicting patient outcomes. However, the mechanism by which *MCTS*1 regulates the tumorigenesis and progression of breast cancer requires further clarification.

## Data Availability

The original contributions presented in the study are included in the article/Supplementary Material, further inquiries can be directed to the corresponding author.

## Footnotes

1
https://portal.gdc.cancer.gov/

2
https://commonfund.nih.gov/gtex

3
https://xenabrowser.net/datapages/

4
http://string-db.org/

5
https://cytoscape.org/

6
http://ualcan.path.uab.edu

7
https://biit.cs.ut.ee/methsurv/

8
https://cran.r-project.org/web/packages/rms/index.html

9
https://www.r-project.org/

## Abbreviations

DSS, disease-specific survival; MCTS1, Multiple copies in T-cell lymphoma-1; OS, overall survival; OR, odds ratio; HR, hazard ratio; CI, confidence interval; C-index, concordance index; TCGA, The Cancer Genome Atlas; GTEx, Genotype Tissue Expression Project; DEG, differentially expressed gene; GO, Gene Ontology; KEGG, Kyoto Encyclopedia of Genes and Genomes; GSEA, gene set enrichment analysis; FDR, false discovery rate; NES, normalized enrichment score; PPI, protein–protein interaction; ROC, receiver operating characteristics; AUC, area under the curve; ER, estrogen receptor; PR, progesterone receptor; HER2, human epidermal growth factor receptor 2; IDC, infiltrating ductal carcinoma; TEM cells, effector T cells; pDCs, plasmacytoid dendritic cells; NK cells, natural killer cells; LumA, Luminal A; LumB, Luminal B; IHC, immunohistochemistry.
