# Supplementary material for: MCTS1 as a Novel Prognostic Biomarker and Its Correlation With Immune Infiltrates in Breast Cancer
Source: Front Genet. 2022 Feb 28;13:825901. doi: 10.3389/fgene.2022.825901 (PMC8918534; doi:10.3389/fgene.2022.825901)
Supplement: Supplementary file 1 [file Table1.DOCX]

Supplementary Material

#
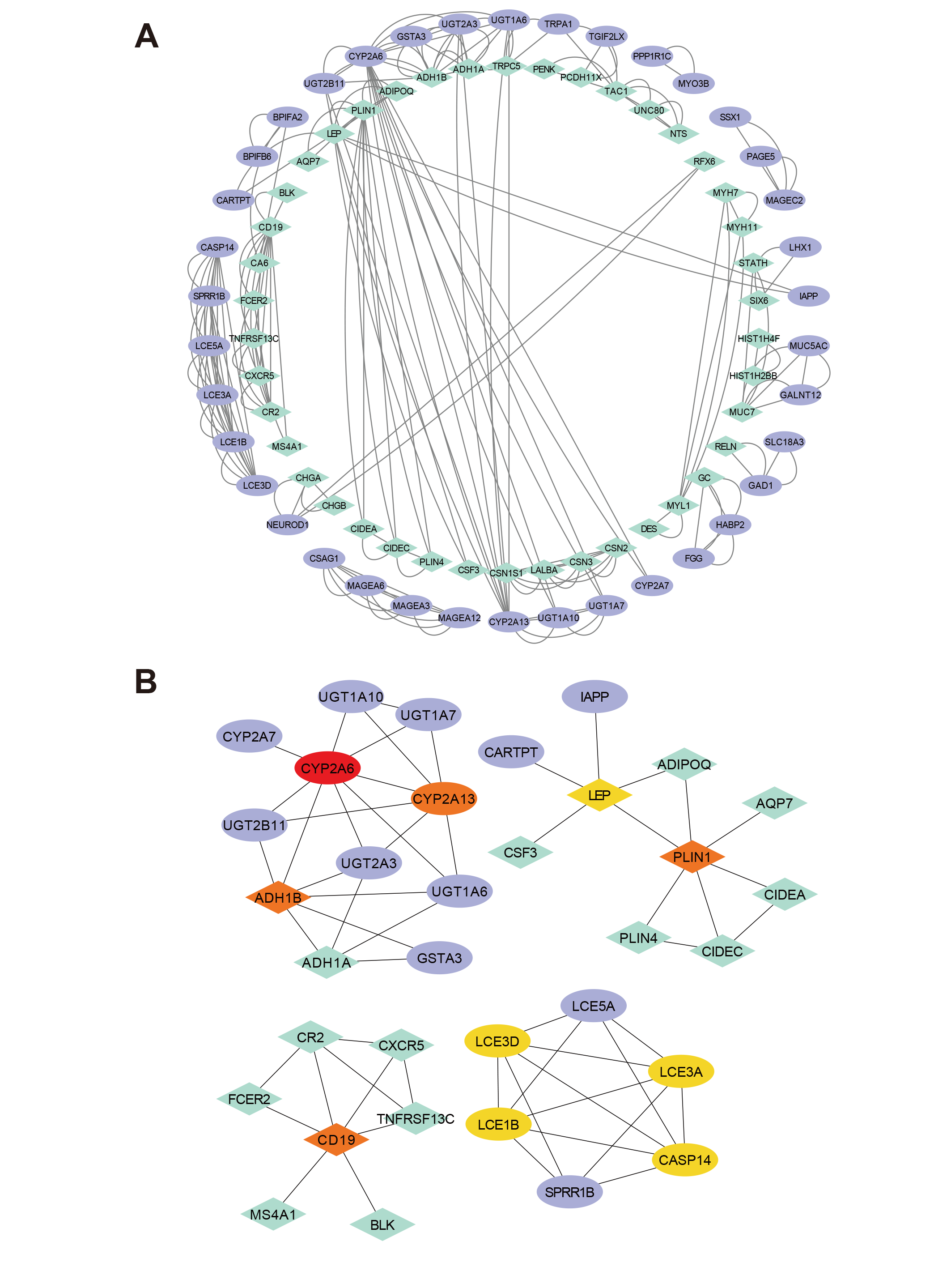


**Supplementary Figure 1.** Protein–protein interaction network of *MCTS1*-related DEGs and top 10 hub genes. **(A)** Protein–protein interaction network of *MCTS1*-related DEGs. Purple ovals and green diamonds represent upregulated and downregulated DEGs, respectively. **(B)** Top 10 hub genes in *MCTS1* expression-associated DEGs. Red to yellow, higher to lower order. DEGs, differentially expressed genes.
