# Supplementary material for: MCTS1 as a Novel Prognostic Biomarker and Its Correlation With Immune Infiltrates in Breast Cancer
Source: Front Genet. 2022 Feb 28;13:825901. doi: 10.3389/fgene.2022.825901 (PMC8918534; doi:10.3389/fgene.2022.825901)
Supplement: Supplementary file 2 [file Table8.DOCX]

Supplementary Material

**Supplementary Table 5**. Univariate and multivariate analyses of overall survival in patients with breast cancer.

| **Characteristics** | **Total (N)** | **Univariate analysis** | |  | **Multivariate analysis** | |
| --- | --- | --- | --- | --- | --- | --- |
|  |  | **Hazard ratio (95% CI)** | ***p* value** |  | **Hazard ratio (95% CI)** | ***p* value** |
| T stage | 1061 |  |  |  |  |  |
| T1 | 274 | Reference |  |  |  |  |
| T2 | 615 | 1.268 (0.842-1.911) | 0.256 |  | 0.623 (0.202-1.918) | 0.410 |
| T3 | 137 | 1.579 (0.937-2.661) | 0.086 |  | 1.038 (0.286-3.764) | 0.955 |
| T4 | 35 | 3.749 (1.953-7.195) | **<0.001** |  | 3.247 (0.651-16.189) | 0.151 |
| N stage | 1045 |  |  |  |  |  |
| N0 | 507 | Reference |  |  |  |  |
| N1 | 348 | 1.826 (1.232-2.706) | **0.003** |  | 0.738 (0.273-1.994) | 0.550 |
| N2 | 116 | 2.502 (1.472-4.253) | **<0.001** |  | 1.427 (0.271-7.517) | 0.675 |
| N3 | 74 | 4.202 (2.323-7.601) | **<0.001** |  | 3.336 (0.597-18.642) | 0.170 |
| M stage | 909 |  |  |  |  |  |
| M0 | 889 | Reference |  |  |  |  |
| M1 | 20 | 4.327 (2.508-7.465) | **<0.001** |  | 13.072 (1.016-168.232) | **0.049** |
| Pathologic stage | 1041 |  |  |  |  |  |
| Stage I | 179 | Reference |  |  |  |  |
| Stage II | 606 | 1.595 (0.923-2.758) | 0.095 |  | 3.760 (0.724-19.530) | 0.115 |
| Stage III | 238 | 2.966 (1.666-5.280) | **<0.001** |  | 9.059 (0.959-85.620) | 0.054 |
| Stage IV | 18 | 11.568 (5.550-24.113) | **<0.001** |  |  |  |
| Age | 1064 |  |  |  |  |  |
| ≤60 | 588 | Reference |  |  |  |  |
| >60 | 476 | 2.036 (1.468-2.822) | **<0.001** |  | 2.716 (1.289-5.722) | **0.009** |

**Supplementary Table 5**. Univariate and multivariate analyses of overall survival in patients with breast cancer (Continued).

| **Characteristics** | **Total(N)** | **Univariate analysis** | |  | **Multivariate analysis** | |
| --- | --- | --- | --- | --- | --- | --- |
|  |  | **Hazard ratio (95% CI)** | ***p* value** |  | **Hazard ratio (95% CI)** | ***p* value** |
| Histological type | 959 |  |  |  |  |  |
| Infiltrating Ductal Carcinoma | 757 | Reference |  |  |  |  |
| Infiltrating Lobular Carcinoma | 202 | 0.860 (0.546-1.355) | 0.516 |  |  |  |
| ER status | 1014 |  |  |  |  |  |
| Negative | 237 | Reference |  |  |  |  |
| Positive | 777 | 0.704 (0.487-1.017) | 0.062 |  | 0.313 (0.082-1.195) | 0.089 |
| PR status | 1011 |  |  |  |  |  |
| Negative | 338 | Reference |  |  |  |  |
| Positive | 673 | 0.762 (0.541-1.074) | 0.120 |  |  |  |
| HER2 status | 705 |  |  |  |  |  |
| Negative | 548 | Reference |  |  |  |  |
| Positive | 157 | 1.611 (0.981-2.644) | 0.059 |  | 1.036 (0.413-2.596) | 0.941 |
| PAM50 | 1024 |  |  |  |  |  |
| Luminal A | 550 | Reference |  |  |  |  |
| Luminal B | 202 | 1.689 (1.103-2.587) | 0.016 |  | 1.424 (0.621-3.261) | 0.404 |
| Her2 | 82 | 2.292 (1.341-3.919) | **0.002** |  | 0.470 (0.099-2.229) | 0.342 |
| Basal | 190 | 1.187 (0.756-1.864) | 0.457 |  | 0.937 (0.224-3.921) | 0.929 |
| Menopause status | 955 |  |  |  |  |  |
| Pre &Peri | 263 | Reference |  |  |  |  |
| Post | 692 | 2.405 (1.445-4.002) | **<0.001** |  | 3.345 (1.137-9.845) | **0.028** |

**Supplementary Table 5**. Univariate and multivariate analyses of overall survival in patients with breast cancer (Continued).

| **Characteristics** | **Total(N)** | **Univariate analysis** | |  | **Multivariate analysis** | |
| --- | --- | --- | --- | --- | --- | --- |
|  |  | **Hazard ratio (95% CI)** | ***p* value** |  | **Hazard ratio (95% CI)** | ***p* value** |
| Anatomic neoplasm subdivisions | 1064 |  |  |  |  |  |
| Left | 553 | Reference |  |  |  |  |
| Right | 511 | 0.776 (0.559-1.077) | 0.130 |  |  |  |
| Radiation therapy | 971 |  |  |  |  |  |
| No | 432 | Reference |  |  |  |  |
| Yes | 539 | 0.558 (0.381-0.819) | **0.003** |  | 0.510 (0.255-1.022) | 0.058 |
| MCTS1 | 1064 |  |  |  |  |  |
| Low | 532 | Reference |  |  |  |  |
| High | 532 | 2.316 (1.652-3.247) | **<0.001** |  | 2.564 (1.346-4.883) | **0.004** |

Abbreviations: ER, estrogen receptor; PR, progesterone receptor; HER2, human epidermal growth factor receptor 2; CI, confidence interval.
