# Supplementary material for: MCTS1 as a Novel Prognostic Biomarker and Its Correlation With Immune Infiltrates in Breast Cancer
Source: Front Genet. 2022 Feb 28;13:825901. doi: 10.3389/fgene.2022.825901 (PMC8918534; doi:10.3389/fgene.2022.825901)
Supplement: Supplementary file 3 [file Table7.DOCX]

**Supplementary Table 4**. KEGG pathway functional enrichment for *MCTS1*-related DEGs.

| **ONTOLOGY** | **ID** | **Description** | **GeneRatio** | **BgRatio** | ***p* value** | **p.adjust** | ***q* value** | **geneID** | **Count** | **zscore** |
| --- | --- | --- | --- | --- | --- | --- | --- | --- | --- | --- |
| KEGG | hsa00980 | Metabolism of xenobiotics by cytochrome P450 | 11/92 | 77/8076 | 8.21708E-10 | 1.24078E-07 | 1.11579E-07 | UGT2B10/UGT2A3/UGT1A6/UGT2A1/GSTA3/CYP2A13/UGT2B11/UGT1A10/UGT1A7/CYP2A6/ADH1A | 11 | 2.713602101 |
| KEGG | hsa05204 | Chemical carcinogenesis | 11/92 | 82/8076 | 1.64396E-09 | 1.24119E-07 | 1.11616E-07 | UGT2B10/UGT2A3/UGT1A6/UGT2A1/GSTA3/CYP2A13/UGT2B11/UGT1A10/UGT1A7/CYP2A6/ADH1A | 11 | 2.713602101 |
| KEGG | hsa00040 | Pentose and glucuronate interconversions | 8/92 | 34/8076 | 2.96732E-09 | 1.49355E-07 | 1.3431E-07 | UGT2B10/UGT2A3/UGT1A6/UGT2A1/AKR1B10/UGT2B11/UGT1A10/UGT1A7 | 8 | 2.828427125 |
| KEGG | hsa00982 | Drug metabolism - cytochrome P450 | 10/92 | 71/8076 | 5.83942E-09 | 2.20438E-07 | 1.98233E-07 | UGT2B10/UGT2A3/UGT1A6/UGT2A1/GSTA3/UGT2B11/UGT1A10/UGT1A7/CYP2A6/ADH1A | 10 | 2.529822128 |
| KEGG | hsa00053 | Ascorbate and aldarate metabolism | 7/92 | 30/8076 | 3.25084E-08 | 9.81755E-07 | 8.82861E-07 | UGT2B10/UGT2A3/UGT1A6/UGT2A1/UGT2B11/UGT1A10/UGT1A7 | 7 | 2.645751311 |
| KEGG | hsa00830 | Retinol metabolism | 9/92 | 68/8076 | 6.16625E-08 | 1.55184E-06 | 1.39552E-06 | UGT2B10/UGT2A3/UGT1A6/UGT2A1/UGT2B11/UGT1A10/UGT1A7/CYP2A6/ADH1A | 9 | 2.333333333 |
| KEGG | hsa00983 | Drug metabolism - other enzymes | 9/92 | 79/8076 | 2.32456E-07 | 5.0144E-06 | 4.50929E-06 | UGT2B10/UGT2A3/UGT1A6/UGT2A1/GSTA3/UGT2B11/UGT1A10/UGT1A7/CYP2A6 | 9 | 3 |
| KEGG | hsa00860 | Porphyrin and chlorophyll metabolism | 7/92 | 42/8076 | 3.85544E-07 | 7.27715E-06 | 6.54411E-06 | UGT2B10/UGT2A3/UGT1A6/UGT2A1/UGT2B11/UGT1A10/UGT1A7 | 7 | 2.645751311 |
| KEGG | hsa00140 | Steroid hormone biosynthesis | 7/92 | 61/8076 | 5.23064E-06 | 8.77585E-05 | 7.89184E-05 | UGT2B10/UGT2A3/UGT1A6/UGT2A1/UGT2B11/UGT1A10/UGT1A7 | 7 | 2.645751311 |
| KEGG | hsa04976 | Bile secretion | 8/92 | 90/8076 | 7.52916E-06 | 0.00011369 | 0.000102238 | UGT2B10/UGT2A3/UGT1A6/UGT2A1/UGT2B11/UGT1A10/UGT1A7/ATP1A2 | 8 | 2.121320344 |
| KEGG | hsa03320 | PPAR signaling pathway | 6/92 | 78/8076 | 0.00024649 | 0.00338363 | 0.00304279 | PCK1/FABP7/AQP7/PLIN1/PLIN4/ADIPOQ | 6 | -2.449489743 |
| KEGG | hsa04080 | Neuroactive ligand-receptor interaction | 12/92 | 341/8076 | 0.000456579 | 0.005745289 | 0.005166555 | IAPP/GABRQ/TAC1/CHRNA4/EDN3/NTS/GLRA3/LEP/UCN3/PENK/NPY2R/CNR2 | 12 | -2.309401077 |
| KEGG | hsa04950 | Maturity onset diabetes of the young | 3/92 | 26/8076 | 0.003076882 | 0.035739167 | 0.03213909 | IAPP/NEUROD1/RFX6 | 3 | 0.577350269 |
| KEGG | hsa04640 | Hematopoietic cell lineage | 5/92 | 99/8076 | 0.005303207 | 0.057198879 | 0.051437123 | FCER2/CSF3/CR2/MS4A1/CD19 | 5 | -2.236067977 |
| KEGG | hsa04920 | Adipocytokine signaling pathway | 4/92 | 69/8076 | 0.007757586 | 0.078093031 | 0.070226567 | IRS4/PCK1/LEP/ADIPOQ | 4 | -1 |

Abbreviations: KEGG, Kyoto Encyclopedia of Genes and Genomes; DEGs, differentially expressed genes.
