# Supplementary material for: MCTS1 as a Novel Prognostic Biomarker and Its Correlation With Immune Infiltrates in Breast Cancer
Source: Front Genet. 2022 Feb 28;13:825901. doi: 10.3389/fgene.2022.825901 (PMC8918534; doi:10.3389/fgene.2022.825901)
Supplement: Supplementary file 4 [file Table9.DOCX]

Supplementary Material

**Supplementary Table 6**. Univariate and multivariate analyses of disease-specific survival in patients with breast cancer.

| **Characteristics** | **Total (N)** | **Univariate analysis** | |  | **Multivariate analysis** | |
| --- | --- | --- | --- | --- | --- | --- |
|  |  | **Hazard ratio (95% CI)** | ***p* value** |  | **Hazard ratio (95% CI)** | ***p* value** |
| T stage | 1042 |  |  |  |  |  |
| T1 | 272 | Reference |  |  |  |  |
| T2 | 605 | 1.437 (0.807-2.557) | 0.218 |  | 0.897 (0.357-2.256) | 0.818 |
| T3 | 131 | 1.790 (0.859-3.729) | 0.120 |  | 0.219 (0.049-0.986) | **0.048** |
| T4 | 34 | 6.745 (3.020-15.068) | **<0.001** |  | 0.517 (0.118-2.272) | 0.383 |
| N stage | 1027 |  |  |  |  |  |
| N0 | 504 | Reference |  |  |  |  |
| N1 | 339 | 3.099 (1.745-5.504) | **<0.001** |  | 1.745 (0.731-4.162) | 0.210 |
| N2 | 112 | 3.724 (1.742-7.961) | **<0.001** |  | 2.680 (0.554-12.972) | 0.220 |
| N3 | 72 | 7.134 (3.321-15.325) | **<0.001** |  | 5.302 (1.272-22.104) | **0.022** |
| M stage | 891 |  |  |  |  |  |
| M0 | 872 | Reference |  |  |  |  |
| M1 | 19 | 7.697 (4.112-14.407) | **<0.001** |  | 10.452 (1.230-88.798) | **0.032** |
| Pathologic stage | 1024 |  |  |  |  |  |
| Stage I | 177 | Reference |  |  |  |  |
| Stage II | 599 | 1.999 (0.835-4.782) | 0.120 |  | 1.563 (0.363-6.727) | 0.548 |
| Stage III | 230 | 5.028 (2.074-12.190) | **<0.001** |  | 2.965 (0.425-20.691) | 0.273 |
| Stage IV | 18 | 26.291 (9.835-70.286) | **<0.001** |  |  |  |
| Age | 1045 |  |  |  |  |  |
| ≤60 | 578 | Reference |  |  |  |  |
| >60 | 467 | 1.418 (0.913-2.201) | 0.120 |  |  |  |

**Supplementary Table 6**. Univariate and multivariate analyses of disease-specific survival in patients with breast cancer (Continued).

| **Characteristics** | **Total(N)** | **Univariate analysis** | |  | **Multivariate analysis** | |
| --- | --- | --- | --- | --- | --- | --- |
|  |  | **Hazard ratio (95% CI)** | ***p* value** |  | **Hazard ratio (95% CI)** | ***p* value** |
| Histological type | 941 |  |  |  |  |  |
| Infiltrating Ductal Carcinoma | 744 | Reference |  |  |  |  |
| Infiltrating Lobular Carcinoma | 197 | 0.500 (0.239-1.045) | 0.065 |  | 0.895 (0.306-2.615) | 0.839 |
| ER status | 996 |  |  |  |  |  |
| Negative | 230 | Reference |  |  |  |  |
| Positive | 766 | 0.523 (0.326-0.838) | **0.007** |  | 0.446 (0.162-1.228) | 0.118 |
| PR status | 993 |  |  |  |  |  |
| Negative | 331 | Reference |  |  |  |  |
| Positive | 662 | 0.529 (0.336-0.833) | **0.006** |  | 0.613 (0.229-1.645) | 0.331 |
| HER2 status | 695 |  |  |  |  |  |
| Negative | 541 | Reference |  |  |  |  |
| Positive | 154 | 1.481 (0.740-2.965) | 0.267 |  |  |  |
| PAM50 | 1006 |  |  |  |  |  |
| Luminal A | 543 | Reference |  |  |  |  |
| Luminal B | 197 | 1.756 (0.974-3.168) | 0.061 |  | 0.754 (0.322-1.764) | 0.515 |
| Her2 | 80 | 2.890 (1.455-5.742) | **0.002** |  | 0.945 (0.302-2.962) | 0.923 |
| Basal | 186 | 1.729 (0.979-3.055) | 0.059 |  | 0.741 (0.228-2.410) | 0.618 |
| Menopause status | 945 |  |  |  |  |  |
| Pre &Peri | 261 | Reference |  |  |  |  |
| Post | 684 | 1.591 (0.872-2.904) | 0.130 |  |  |  |

**Supplementary Table 6**. Univariate and multivariate analyses of disease-specific survival in patients with breast cancer (Continued).

| **Characteristics** | **Total(N)** | **Univariate analysis** | |  | **Multivariate analysis** | |
| --- | --- | --- | --- | --- | --- | --- |
|  |  | **Hazard ratio (95% CI)** | ***p* value** |  | **Hazard ratio (95% CI)** | ***p* value** |
| Anatomic neoplasm subdivisions | 1045 |  |  |  |  |  |
| Left | 541 | Reference |  |  |  |  |
| Right | 504 | 0.797 (0.513-1.239) | 0.314 |  |  |  |
| Radiation therapy | 962 |  |  |  |  |  |
| No | 428 | Reference |  |  |  |  |
| Yes | 534 | 0.755 (0.458-1.244) | 0.271 |  |  |  |
| MCTS1 | 1045 |  |  |  |  |  |
| Low | 523 | Reference |  |  |  |  |
| High | 522 | 2.520 (1.586-4.005) | **<0.001** |  | 3.267 (1.723-6.195) | **<0.001** |

Abbreviations: ER, estrogen receptor; PR, progesterone receptor; HER2, human epidermal growth factor receptor 2; CI, confidence interval.
