# Supplementary material for: MCTS1 as a Novel Prognostic Biomarker and Its Correlation With Immune Infiltrates in Breast Cancer
Source: Front Genet. 2022 Feb 28;13:825901. doi: 10.3389/fgene.2022.825901 (PMC8918534; doi:10.3389/fgene.2022.825901)
Supplement: Supplementary file 5 [file Table2.DOCX]

Supplementary Material


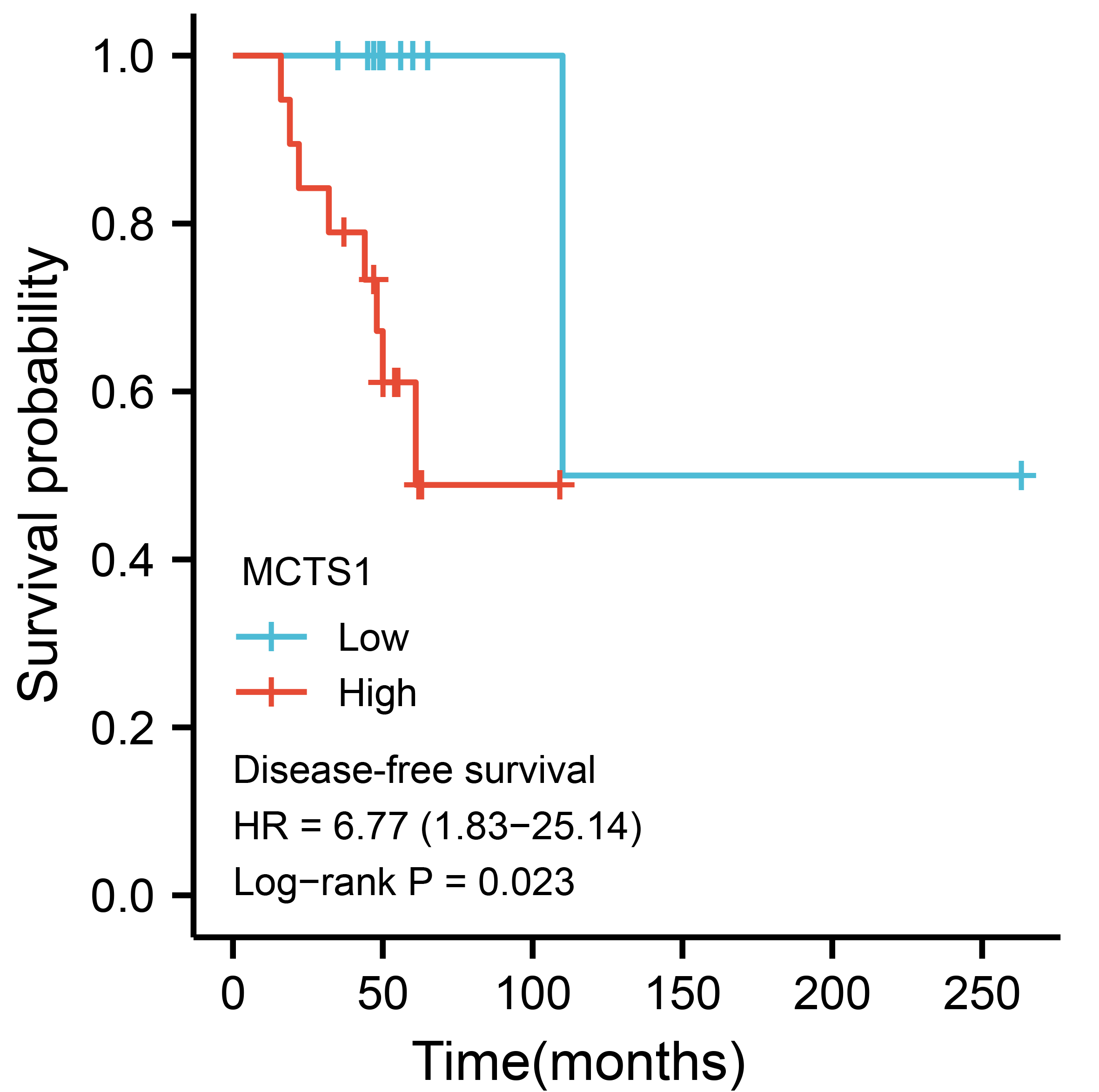


**Supplementary figure 2.** Disease-free survival for patients with breast cancer.
