# Supplementary material for: MCTS1 as a Novel Prognostic Biomarker and Its Correlation With Immune Infiltrates in Breast Cancer
Source: Front Genet. 2022 Feb 28;13:825901. doi: 10.3389/fgene.2022.825901 (PMC8918534; doi:10.3389/fgene.2022.825901)
Supplement: Supplementary file 6 [file Table5.DOCX]

Supplementary Material

**Supplementary Table 2**. Top 10 upregulated and 10 downregulated *MCTS1*-related DEGs.

| **gene_name** | **gene_id** | **gene_biotype** | **baseMean** | **log2FoldChange** | **lfcSE** | **stat** | ***p* value** | **p.adj** |
| --- | --- | --- | --- | --- | --- | --- | --- | --- |
| KLHL1 | ENSG00000150361 | protein_coding | 31.32338563 | 2.876709585 | 0.404945865 | 7.103936194 | 1.21253E-12 | 2.29721E-11 |
| CARTPT | ENSG00000164326 | protein_coding | 1122.681668 | 2.385762587 | 0.388529324 | 6.140495554 | 8.22644E-10 | 8.78213E-09 |
| IAPP | ENSG00000121351 | protein_coding | 1.047514709 | 2.375460793 | 0.447464523 | 5.308713145 | 1.10402E-07 | 7.71114E-07 |
| S100G | ENSG00000169906 | protein_coding | 26.60680061 | 2.330589514 | 0.250183929 | 9.315504477 | 1.21374E-20 | 1.14926E-18 |
| MAGEB16 | ENSG00000189023 | protein_coding | 0.826649022 | 2.17945146 | 0.403778439 | 5.397641991 | 6.75224E-08 | 4.93545E-07 |
| PAGE2B | ENSG00000238269 | protein_coding | 4.952205897 | 2.111600815 | 0.191812864 | 11.00865067 | 3.47164E-28 | 1.41318E-25 |
| IRS4 | ENSG00000133124 | protein_coding | 16.64200945 | 2.069613683 | 0.228765245 | 9.046888591 | 1.471E-19 | 1.10277E-17 |
| MAGEA12 | ENSG00000213401 | protein_coding | 38.69908499 | 2.067344401 | 0.323312486 | 6.394260933 | 1.61326E-10 | 1.96551E-09 |
| PPP4R3C | ENSG00000224960 | protein_coding | 0.710782112 | 1.998081023 | 0.482337199 | 4.142498291 | 3.43543E-05 | 0.000141417 |
| UGT2B11 | ENSG00000213759 | protein_coding | 1427.746815 | 1.99654132 | 0.231295959 | 8.631976673 | 6.03007E-18 | 3.25057E-16 |
| CSN2 | ENSG00000135222 | protein_coding | 96.4623941 | -7.407910017 | 0.491015504 | -15.08691672 | 1.97456E-51 | 7.167E-48 |
| LALBA | ENSG00000167531 | protein_coding | 90.24493384 | -4.174520268 | 0.432732411 | -9.646886074 | 5.0671E-22 | 6.09676E-20 |
| SMR3B | ENSG00000171201 | protein_coding | 154.3726503 | -3.486990827 | 0.345385866 | -10.09592798 | 5.75838E-24 | 1.01134E-21 |
| SMR3A | ENSG00000109208 | protein_coding | 1.786457984 | -3.090325877 | 0.51281744 | -6.026171565 | 1.67889E-09 | 1.68337E-08 |
| CHGA | ENSG00000100604 | protein_coding | 430.5798369 | -2.262995268 | 0.223407299 | -10.12945986 | 4.08902E-24 | 7.42089E-22 |
| FGF4 | ENSG00000075388 | protein_coding | 3.57790053 | -2.203700247 | 0.439694598 | -5.011888384 | 5.38985E-07 | 3.25694E-06 |
| BPIFB3 | ENSG00000186190 | protein_coding | 0.717647546 | -2.124699137 | 0.519855006 | -4.087099507 | 4.368E-05 | 0.000175785 |
| NTS | ENSG00000133636 | protein_coding | 165.1714313 | -2.051381523 | 0.244430393 | -8.392497756 | 4.75921E-17 | 2.1239E-15 |
| CSN1S1 | ENSG00000126545 | protein_coding | 15.90277426 | -1.88964315 | 0.254781334 | -7.416725244 | 1.20052E-13 | 2.80223E-12 |
| STATH | ENSG00000126549 | protein_coding | 0.701358903 | -1.818967083 | 0.588971023 | -3.08838128 | 0.002012501 | 0.005510214 |

Abbreviations: DEGs, differentially expressed genes; p.adj, adjusted *p* value.
