# Supplementary material for: MCTS1 as a Novel Prognostic Biomarker and Its Correlation With Immune Infiltrates in Breast Cancer
Source: Front Genet. 2022 Feb 28;13:825901. doi: 10.3389/fgene.2022.825901 (PMC8918534; doi:10.3389/fgene.2022.825901)
Supplement: Supplementary file 7 [file Table3.DOCX]

Supplementary Material


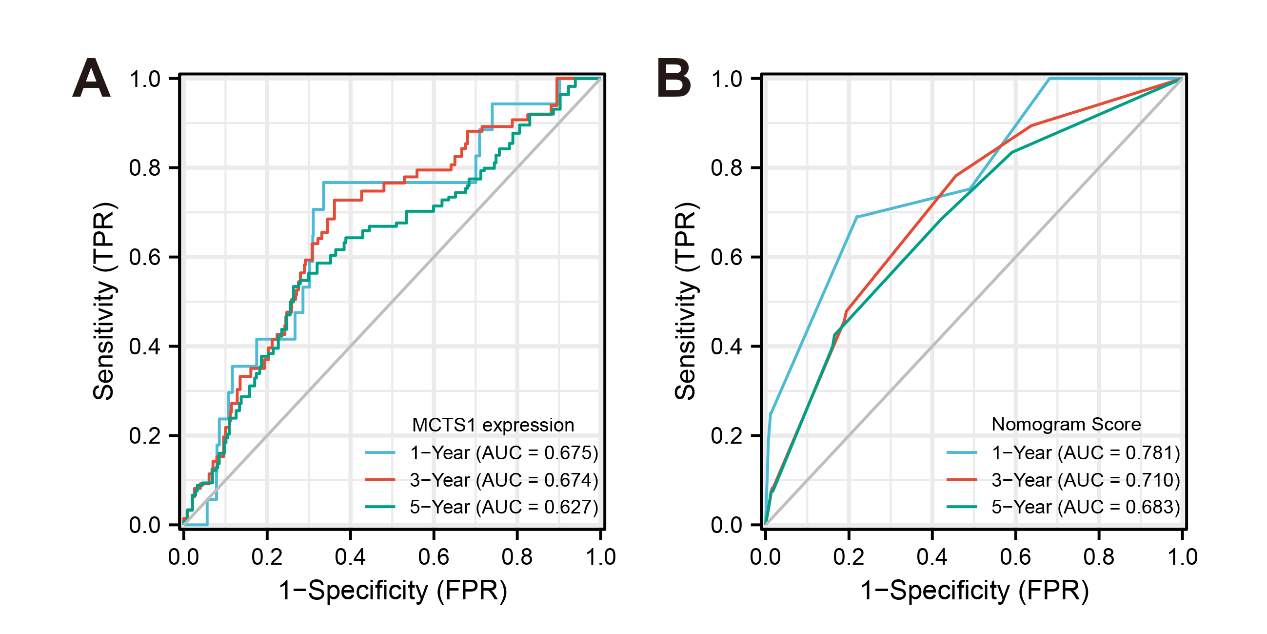


**Supplementary figure 3.** Time-dependent ROC curves at different time points.

Time-dependent ROC curves based on **(A)** *MCTS1* expression and (B) nomogram score for one-, three-, and five-year OS probability. ROC, receiver-operating characteristic; AUC, area under the ROC curve; OS, overall survival.
