# Supplementary material for: MCTS1 as a Novel Prognostic Biomarker and Its Correlation With Immune Infiltrates in Breast Cancer
Source: Front Genet. 2022 Feb 28;13:825901. doi: 10.3389/fgene.2022.825901 (PMC8918534; doi:10.3389/fgene.2022.825901)
Supplement: Supplementary file 8 [file Table4.DOCX]

Supplementary Material

**Supplementary Table 1**. Baseline characteristics of patients (n = 1,065).

| **Characteristic** | **levels** | **Overall** |
| --- | --- | --- |
| n |  | 1065 |
| Age, median (IQR) |  | 58 (49, 67) |
| Age, n (%) | ≤60 | 588 (55.2%) |
|  | >60 | 477 (44.8%) |
| T stage, n (%) | T1 | 275 (25.9%) |
|  | T2 | 615 (57.9%) |
|  | T3 | 137 (12.9%) |
|  | T4 | 35 (3.3%) |
| N stage, n (%) | N0 | 507 (48.5%) |
|  | N1 | 349 (33.4%) |
|  | N2 | 116 (11.1%) |
|  | N3 | 74 (7.1%) |
| M stage, n (%) | M0 | 889 (97.8%) |
|  | M1 | 20 (2.2%) |
| Pathologic stage, n (%) | Stage I | 180 (17.3%) |
|  | Stage II | 606 (58.2%) |
|  | Stage III | 238 (22.8%) |
|  | Stage IV | 18 (1.7%) |
| Race, n (%) | Asian | 60 (6.1%) |
|  | Black or African American | 179 (18.3%) |
|  | White | 737 (75.5%) |
| Histological type, n (%) | Infiltrating Ductal Carcinoma | 757 (78.9%) |
|  | Infiltrating Lobular Carcinoma | 202 (21.1%) |
| PR status, n (%) | Negative | 338 (33.3%) |
|  | Indeterminate | 4 (0.4%) |
|  | Positive | 674 (66.3%) |
| ER status, n (%) | Negative | 237 (23.3%) |
|  | Indeterminate | 2 (0.2%) |
|  | Positive | 778 (76.5%) |
| HER2 status, n (%) | Negative | 548 (76.4%) |
|  | Indeterminate | 12 (1.7%) |
|  | Positive | 157 (21.9%) |
| PAM50, n (%) | Normal | 40 (3.8%) |
|  | Luminal A | 551 (51.7%) |
|  | Luminal B | 202 (19%) |

**Supplementary Table 1**. Baseline characteristics of patients (n = 1,065) (Continued).

| **Characteristic** | **levels** | **Overall** |
| --- | --- | --- |
|  | Her2 | 82 (7.7%) |
|  | Basal | 190 (17.8%) |
| Menopause status, n (%) | Pre | 224 (23.4%) |
|  | Peri | 39 (4.1%) |
|  | Post | 693 (72.5%) |
| Anatomic neoplasm subdivisions, n (%) | Left | 553 (51.9%) |
|  | Right | 512 (48.1%) |
| Radiation therapy, n (%) | No | 432 (44.4%) |
|  | Yes | 540 (55.6%) |
| OS event, n (%) | Alive | 918 (86.2%) |
|  | Dead | 147 (13.8%) |
| DSS event, n (%) | Alive | 965 (92.3%) |
|  | Dead | 81 (7.7%) |

Abbreviations: ER, estrogen receptor; PR, progesterone receptor; HER2, human epidermal growth factor receptor 2; OS, overall survival; DSS, disease-specific survival.
