# Supplementary material for: MCTS1 as a Novel Prognostic Biomarker and Its Correlation With Immune Infiltrates in Breast Cancer
Source: Front Genet. 2022 Feb 28;13:825901. doi: 10.3389/fgene.2022.825901 (PMC8918534; doi:10.3389/fgene.2022.825901)
Supplement: Supplementary file 9 [file Table6.DOCX]

Supplementary Material

**Supplementary Table 3**. Gene Ontology pathway functional enrichment for *MCTS1*-related DEGs.

| **ONTOLOGY** | **ID** | **Description** | **GeneRatio** | **BgRatio** | ***p* value** | **p.adjust** | ***q* value** | **geneID** | **Count** | **zscore** |
| --- | --- | --- | --- | --- | --- | --- | --- | --- | --- | --- |
| BP | GO:0052695 | cellular glucuronidation | 6/187 | 19/18670 | 2.26851E-08 | 5.38545E-05 | 4.97402E-05 | UGT2A3/UGT1A6/UGT2A1/UGT2B11/UGT1A10/UGT1A7 | 6 | 2.449489743 |
| BP | GO:0006063 | uronic acid metabolic process | 6/187 | 24/18670 | 1.07945E-07 | 8.54203E-05 | 7.88944E-05 | UGT2A3/UGT1A6/UGT2A1/UGT2B11/UGT1A10/UGT1A7 | 6 | 2.449489743 |
| BP | GO:0019585 | glucuronate metabolic process | 6/187 | 24/18670 | 1.07945E-07 | 8.54203E-05 | 7.88944E-05 | UGT2A3/UGT1A6/UGT2A1/UGT2B11/UGT1A10/UGT1A7 | 6 | 2.449489743 |
| BP | GO:0009410 | response to xenobiotic stimulus | 15/187 | 292/18670 | 2.64074E-07 | 0.000156728 | 0.000144754 | BCHE/UGT1A6/GSTA3/CYP2A13/CYP2A7/UGT2B11/UGT1A10/UGT1A7/CYP2A6/TAC1/PCK1/FOSB/GLYAT/PENK/CNR2 | 15 | 0.774596669 |
| BP | GO:0006805 | xenobiotic metabolic process | 10/187 | 125/18670 | 5.23242E-07 | 0.000248435 | 0.000229456 | BCHE/UGT1A6/GSTA3/CYP2A13/CYP2A7/UGT2B11/UGT1A10/UGT1A7/CYP2A6/GLYAT | 10 | 2.529822128 |
| BP | GO:0019233 | sensory perception of pain | 9/187 | 104/18670 | 1.01425E-06 | 0.000401306 | 0.000370647 | TRPA1/IAPP/TAC1/CHRNA4/SMR3A/SMR3B/PENK/NPY2R/CNR2 | 9 | -1.666666667 |
| BP | GO:0071466 | cellular response to xenobiotic stimulus | 11/187 | 180/18670 | 2.05945E-06 | 0.000698448 | 0.000645088 | BCHE/UGT1A6/GSTA3/CYP2A13/CYP2A7/UGT2B11/UGT1A10/UGT1A7/CYP2A6/PCK1/GLYAT | 11 | 2.110579412 |
| BP | GO:0052697 | xenobiotic glucuronidation | 4/187 | 11/18670 | 3.04441E-06 | 0.000903429 | 0.000834409 | UGT1A6/UGT2B11/UGT1A10/UGT1A7 | 4 | 2 |
| BP | GO:0046887 | positive regulation of hormone secretion | 9/187 | 131/18670 | 6.92284E-06 | 0.001826091 | 0.001686582 | CARTPT/SLC30A8/FGG/TAC1/EDN3/BLK/LEP/UCN3/RFX6 | 9 | -1 |
| BP | GO:0046883 | regulation of hormone secretion | 12/187 | 266/18670 | 1.59985E-05 | 0.003798038 | 0.003507876 | NEUROD1/CARTPT/SLC30A8/FGG/TAC1/CHGA/EDN3/BLK/LEP/UCN3/ADIPOQ/RFX6 | 12 | -1.154700538 |
| BP | GO:0018149 | peptide cross-linking | 6/187 | 60/18670 | 2.97723E-05 | 0.006097321 | 0.005631499 | LCE3D/SPRR1B/LCE3A/LCE5A/LCE1B/KRT1 | 6 | 1.632993162 |
| BP | GO:0042391 | regulation of membrane potential | 15/187 | 434/18670 | 3.30288E-05 | 0.006097321 | 0.005631499 | KCNK16/SLC1A6/CACNG2/GABRQ/TAC1/ATP1A2/TRPC5/CHRNA4/KCNH5/GLRA3/UCN3/KCNH8/NPY2R/CNR2/RELN | 15 | -1.807392228 |
| BP | GO:0031640 | killing of cells of other organism | 6/187 | 62/18670 | 3.59572E-05 | 0.006097321 | 0.005631499 | DEFB4A/LCE3A/CHGA/FCER2/DCD/MUC7 | 6 | -0.816496581 |
| BP | GO:0044364 | disruption of cells of other organism | 6/187 | 62/18670 | 3.59572E-05 | 0.006097321 | 0.005631499 | DEFB4A/LCE3A/CHGA/FCER2/DCD/MUC7 | 6 | -0.816496581 |
| BP | GO:0023061 | signal release | 15/187 | 462/18670 | 6.71151E-05 | 0.010622081 | 0.009810577 | GAD1/NEUROD1/CARTPT/SLC30A8/FGG/SLC18A3/TAC1/CHGA/CHRNA4/EDN3/BLK/LEP/UCN3/ADIPOQ/RFX6 | 15 | -0.774596669 |
| BP | GO:0046879 | hormone secretion | 12/187 | 312/18670 | 7.56097E-05 | 0.011218595 | 0.010361519 | NEUROD1/CARTPT/SLC30A8/FGG/TAC1/CHGA/EDN3/BLK/LEP/UCN3/ADIPOQ/RFX6 | 12 | -1.154700538 |
| BP | GO:0009914 | hormone transport | 12/187 | 322/18670 | 0.000101933 | 0.014234623 | 0.013147129 | NEUROD1/CARTPT/SLC30A8/FGG/TAC1/CHGA/EDN3/BLK/LEP/UCN3/ADIPOQ/RFX6 | 12 | -1.154700538 |
| BP | GO:0072503 | cellular divalent inorganic cation homeostasis | 15/187 | 493/18670 | 0.000137735 | 0.018165731 | 0.016777909 | TRPA1/IAPP/SLC30A8/CCL15/TAC1/ATP1A2/TRPC5/EDN3/GPR12/LACRT/GPR17/MS4A1/CXCR5/CD19/NPY2R | 15 | -1.807392228 |
|  |  | cellular calcium ion homeostasis | 14/187 | 458/18670 | 0.000219941 | 0.026146226 | 0.024148712 | TRPA1/IAPP/CCL15/TAC1/ATP1A2/TRPC5/EDN3/GPR12/LACRT/GPR17/MS4A1/CXCR5/CD19/NPY2R |  |  |
| BP | GO:0030072 | peptide hormone secretion | 10/187 | 250/18670 | 0.000220271 | 0.026146226 | 0.024148712 | NEUROD1/CARTPT/SLC30A8/FGG/CHGA/EDN3/BLK/LEP/UCN3/RFX6 | 10 | -0.632455532 |
| BP | GO:0090276 | regulation of peptide hormone secretion | 9/187 | 208/18670 | 0.000255409 | 0.027889072 | 0.025758408 | NEUROD1/CARTPT/SLC30A8/FGG/CHGA/BLK/LEP/UCN3/RFX6 | 9 | -0.333333333 |
| BP | GO:0006959 | humoral immune response | 12/187 | 356/18670 | 0.00025845 | 0.027889072 | 0.025758408 | BPIFA2/DEFB4A/CHGA/FCER2/C7/CR2/IFNK/MS4A1/DCD/KRT1/MUC7/CD19 | 12 | -2.309401077 |

**Supplementary Table 3**. Gene Ontology pathway functional enrichment for *MCTS1*-related DEGs (Continued).

| **ONTOLOGY** | **ID** | **Description** | **GeneRatio** | **BgRatio** | ***p* value** | **p.adjust** | ***q* value** | **geneID** | **Count** | **zscore** |
| --- | --- | --- | --- | --- | --- | --- | --- | --- | --- | --- |
| BP | GO:0055074 | calcium ion homeostasis | 14/187 | 471/18670 | 0.000292322 | 0.030172715 | 0.027867586 | TRPA1/IAPP/CCL15/TAC1/ATP1A2/TRPC5/EDN3/GPR12/LACRT/GPR17/MS4A1/CXCR5/CD19/NPY2R | 14 | -2.138089935 |
| BP | GO:0021527 | spinal cord association neuron differentiation | 3/187 | 14/18670 | 0.000331816 | 0.032822161 | 0.03031462 | LBX1/LHX1/GDF7 | 3 | 0.577350269 |
| BP | GO:0007204 | positive regulation of cytosolic calcium ion concentration | 11/187 | 319/18670 | 0.000385254 | 0.034907935 | 0.032241045 | TRPA1/IAPP/TAC1/ATP1A2/TRPC5/LACRT/GPR17/MS4A1/CXCR5/CD19/NPY2R | 11 | -2.110579412 |
| BP | GO:0051952 | regulation of amine transport | 6/187 | 95/18670 | 0.000387028 | 0.034907935 | 0.032241045 | CARTPT/ATP1A2/CHGA/CHRNA4/LEP/NPY2R | 6 | -1.632993162 |
| BP | GO:0050796 | regulation of insulin secretion | 8/187 | 176/18670 | 0.000405236 | 0.034907935 | 0.032241045 | NEUROD1/CARTPT/SLC30A8/CHGA/BLK/LEP/UCN3/RFX6 | 8 | -0.707106781 |
| BP | GO:0009812 | flavonoid metabolic process | 3/187 | 15/18670 | 0.00041172 | 0.034907935 | 0.032241045 | UGT1A6/UGT1A10/UGT1A7 | 3 | 1.732050808 |
| BP | GO:0051591 | response to cAMP | 6/187 | 97/18670 | 0.000432856 | 0.035434516 | 0.032727396 | PCK1/FOSB/GPD1/AGXT/ADIPOQ/PENK | 6 | -2.449489743 |
| BP | GO:0019748 | secondary metabolic process | 5/187 | 67/18670 | 0.000558232 | 0.043347925 | 0.040036238 | CYP2A13/AKR1B10/CYP2A7/UGT1A7/CYP2A6 | 5 | 2.236067977 |
| BP | GO:0015837 | amine transport | 6/187 | 102/18670 | 0.000566043 | 0.043347925 | 0.040036238 | CARTPT/ATP1A2/CHGA/CHRNA4/LEP/NPY2R | 6 | -1.632993162 |
| BP | GO:0005996 | monosaccharide metabolic process | 10/187 | 292/18670 | 0.000745653 | 0.055318107 | 0.051091924 | UGT2A3/UGT1A6/UGT2A1/UGT2B11/UGT1A10/UGT1A7/PCK1/GPD1/LEP/ADIPOQ | 10 | 0.632455532 |
| BP | GO:0033555 | multicellular organismal response to stress | 5/187 | 73/18670 | 0.000827259 | 0.0595125 | 0.054965875 | TRPA1/TAC1/PENK/NPY2R/RELN | 5 | -1.341640786 |
| BP | GO:0007626 | locomotory behavior | 8/187 | 198/18670 | 0.000879274 | 0.061393989 | 0.056703622 | GAD1/KLHL1/ATP1A2/CHRNA4/NKX2-1/FEZF2/PENK/NPY2R | 8 | -1.414213562 |
| BP | GO:0051480 | regulation of cytosolic calcium ion concentration | 11/187 | 357/18670 | 0.000976154 | 0.065563862 | 0.060554926 | TRPA1/IAPP/TAC1/ATP1A2/TRPC5/LACRT/GPR17/MS4A1/CXCR5/CD19/NPY2R | 11 | -2.110579412 |
| BP | GO:0019373 | epoxygenase P450 pathway | 3/187 | 20/18670 | 0.000994229 | 0.065563862 | 0.060554926 | CYP2A13/CYP2A7/CYP2A6 | 3 | 1.732050808 |
| BP | GO:0034389 | lipid droplet organization | 3/187 | 21/18670 | 0.001151426 | 0.073120617 | 0.067534362 | HSD17B13/CIDEA/CIDEC | 3 | -1.732050808 |
| BP | GO:0030073 | insulin secretion | 8/187 | 207/18670 | 0.001170423 | 0.073120617 | 0.067534362 | NEUROD1/CARTPT/SLC30A8/CHGA/BLK/LEP/UCN3/RFX6 | 8 | -0.707106781 |
| BP | GO:0031960 | response to corticosteroid | 7/187 | 162/18670 | 0.00125038 | 0.076112873 | 0.070298016 | BCHE/PCK1/FOSB/CSN1S1/AGXT/UCN3/ADIPOQ | 7 | -1.889822365 |
| BP | GO:0021516 | dorsal spinal cord development | 3/187 | 22/18670 | 0.001323455 | 0.078547081 | 0.072546255 | LBX1/LHX1/GDF7 | 3 | 0.577350269 |
| BP | GO:0089718 | amino acid import across plasma membrane | 3/187 | 23/18670 | 0.001510821 | 0.085930276 | 0.07936539 | SLC1A6/ATP1A2/SLC7A3 | 3 | -0.577350269 |
| BP | GO:0001906 | cell killing | 7/187 | 168/18670 | 0.001541547 | 0.085930276 | 0.07936539 | DEFB4A/LCE3A/CHGA/FCER2/DCD/MUC7/LEP | 7 | -1.133893419 |
| BP | GO:0097756 | negative regulation of blood vessel diameter | 5/187 | 84/18670 | 0.001556446 | 0.085930276 | 0.07936539 | FGG/ATP1A2/CHGA/EDN3/LEP | 5 | -1.341640786 |
| BP | GO:0021871 | forebrain regionalization | 3/187 | 24/18670 | 0.001714006 | 0.090423339 | 0.083515193 | LHX1/NKX2-1/FEZF2 | 3 | -0.577350269 |
| BP | GO:0042738 | exogenous drug catabolic process | 3/187 | 24/18670 | 0.001714006 | 0.090423339 | 0.083515193 | CYP2A13/CYP2A7/CYP2A6 | 3 | 1.732050808 |
| CC | GO:0001533 | cornified envelope | 7/198 | 65/19717 | 3.93982E-06 | 0.0009101 | 0.0009101 | LCE3D/SPRR1B/LCE3A/LCE5A/LCE1B/FLG2/KRT1 | 7 | 1.133893419 |
| CC | GO:0005811 | lipid droplet | 6/198 | 81/19717 | 0.000165064 | 0.019064836 | 0.019064836 | AQP7/PLIN1/PLIN4/HSD17B13/CIDEA/CIDEC | 6 | -2.449489743 |
| MF | GO:0015020 | glucuronosyltransferase activity | 6/186 | 33/17697 | 1.08772E-06 | 0.000397016 | 0.000368678 | UGT2A3/UGT1A6/UGT2A1/UGT2B11/UGT1A10/UGT1A7 | 6 | 2.449489743 |
| MF | GO:0005179 | hormone activity | 9/186 | 122/17697 | 5.66821E-06 | 0.001034448 | 0.000960612 | IAPP/CARTPT/CHGB/EDN3/NTS/LEP/UCN3/ADIPOQ/PENK | 9 | -1.666666667 |

**Supplementary Table 3**. Gene Ontology pathway functional enrichment for *MCTS1*-related DEGs (Continued).

| **ONTOLOGY** | **ID** | **Description** | **GeneRatio** | **BgRatio** | ***p* value** | **p.adjust** | ***q* value** | **geneID** | **Count** | **zscore** |
| --- | --- | --- | --- | --- | --- | --- | --- | --- | --- | --- |
| MF | GO:0048018 | receptor ligand activity | 17/186 | 482/17697 | 1.38067E-05 | 0.00167982 | 0.001559919 | IAPP/CARTPT/DEFB4A/CCL15/FGF4/CHGB/CSF3/EDN3/NTS/LACRT/GDF7/IFNK/CMTM5/LEP/UCN3/ADIPOQ/PENK | 17 | -2.182820625 |
| MF | GO:0008194 | UDP-glycosyltransferase activity | 8/186 | 151/17697 | 0.000197951 | 0.01806305 | 0.016773762 | UGT2B10/UGT2A3/UGT1A6/UGT2A1/UGT2B11/UGT1A10/UGT1A7/LALBA | 8 | 2.121320344 |
| MF | GO:0008392 | arachidonic acid epoxygenase activity | 3/186 | 16/17697 | 0.000578399 | 0.042223131 | 0.039209368 | CYP2A13/CYP2A7/CYP2A6 | 3 | 1.732050808 |
| MF | GO:0008391 | arachidonic acid monooxygenase activity | 3/186 | 17/17697 | 0.000696928 | 0.042396447 | 0.039370313 | CYP2A13/CYP2A7/CYP2A6 | 3 | 1.732050808 |

Abbreviations: BP, biological processes; CC, subcellular localizations; MF, molecular functions; DEGs, differentially expressed genes.
